# ﻿The genus *Atopsyche* (Trichoptera, Hydrobiosidae) in Peru, with the description of seven new species

**DOI:** 10.3897/zookeys.1263.150396

**Published:** 2025-12-10

**Authors:** Ernesto Rázuri-Gonzales, Ralph W. Holzenthal

**Affiliations:** 1 Senckenberg Research Institute and Natural History Museum Frankfurt, Frankfurt am Main, Germany Senckenberg Research Institute and Natural History Museum Frankfurt Frankfurt am Main Germany; 2 Department of Entomology, University of Minnesota, St. Paul, Minnesota, USA University of Minnesota St. Paul United States of America; 3 Departamento de Entomología, Museo de Historia Natural, Universidad Nacional Mayor de San Marcos, Lima, Peru Universidad Nacional Mayor de San Marcos Lima Peru

**Keywords:** Aquatic insects, caddisfly, morphology, taxonomy, tropical Andes

## Abstract

The caddisfly genus *Atopsyche* (Trichoptera: Hydrobiosidae) in Peru currently includes 12 species, three of which occur only in Peru. Herein, we describe and illustrate seven new species: *A. cedroi***sp. nov.**, *A. chemillen***sp. nov.**, *A. corcuerai***sp. nov.**, *A. huascarani***sp. nov.**, *A. refulioae***sp. nov.**, *A. sofiae***sp. nov.**, and *A. yanachaga***sp. nov.** We also include illustrations for the species included in the subgenus Dolochorema as a comparison. Finally, one new country record is added, *A. tincuracu*, and expand the distributional range of five additional species, thus increasing the number of species of *Atopsyche* from Peru to 20.

## ﻿Introduction

The New World genus *Atopsyche* (Trichoptera: Hydrobiosidae) currently includes 153 extant and one fossil species (Holzenthal and Calor 2017; Zamora-Muñoz et al. 2017; Mey and Ospina-Torres 2018; Gomes and Calor 2019; Calor et al. 2023; Razo-González et al. 2023; Rázuri-Gonzales et al. 2025), and is by far, the most diverse and widespread genus in the family. It occurs in the Brazilian Subregion of the Neotropics, extending into Central America, the Antilles, and the southwestern USA (Holzenthal et al. 2007). The genus is notably absent from the Chilean Subregion, where it is replaced by a suite of other endemic hydrobiosid genera (Holzenthal and Calor 2017).

The genus is divided into three subgenera: the nominotypical *Atopsyche*, *Atopsaura* Ross, 1953, and Dolochorema Banks, 1913. The subgenus Atopsyche has the first segment of the inferior appendages without any projection, while *Atopsaura* has an apical projection that can be dorsal, mesal, or ventral. The subgenus Dolochorema is characterized by the second segment of the inferior appendage having moved to the mesal surface of the first, mostly unseen in lateral view. Additionally, Schmid (1989) established the *bicolorata* species group, not included in any subgenus, and two species of uncertain placement within the genus. The *bicolorata* species group is characterized by having short inferior appendages, which are broadly notched apically. The second segment of the inferior appendage is reduced and inserted on the posterodorsal corner of the first segment or in its notch. Additionally, the apical half of the phallotheca is membranous.

Additionally, Ross (1953) proposed several species groups within these subgenera, but the characters used to separate these species groups occur in representatives of more than one species group. For example, the absence or presence of the ventrolateral branches and the basodorsal processes of the phallic apparatus contradict these groups. Schmid (1989), in his revision of the Hydrobiosidae placed the 45 species he described in Ross’s subgenera. However, he and later Blahnik and Gottschalk (1997) recognized the need for an updated subgeneric classification of this genus.

This paper describes seven new species of *Atopsyche* from various localities along the central and northern Peruvian Andes: *A. cedroi* sp. nov., *A. chemillen* sp. nov., *A. refulioae* sp. nov., and *A. yanachaga* sp. nov. (Pasco Department); *A. huascarani* sp. nov. (Ancash Department); *A. sofiae* sp. nov. (La Libertad Department); and *A. corcuerai* sp. nov. (Cajamarca Department). Additionally, we report a new country record for *Atopsyche tincuracu* Schmid, 1989, and extend the known distribution ranges for five other species. As a result, the total number of *Atopsyche* species recorded from Peru has increased to 20, raising the overall total for the genus to 160 species.

## ﻿Materials and methods

### ﻿Specimen collection, preparation, and observation

Adult specimens were collected using light traps consisting of a 250-watt mixed-light lamp, powered by a gasoline generator. These lamps contain a high-pressure mercury tube and an incandescent element, the former providing a line spectrum in the ultraviolet range (360–440 nm) and the latter a continuous spectrum in the visible and infrared range (500–780 nm). The lights were set up in front of a white sheet suspended by a cord between two trees or stakes and positioned next to stream environments. The traps were typically operated from 6 p.m. to midnight. Collected specimens were killed using a potassium cyanide killing jar or by immersing them in ethanol. The specimens collected with the potassium cyanide killing jar were later pinned using entomological pins.

The specimens were prepared and examined using standard techniques outlined by Blahnik and Holzenthal (2004) and Blahnik et al. (2007). Forewing length was measured from base to apex and reported as an average, along with the number of specimens measured. Male genitalia were macerated with 85% lactic acid at 120 °C until the tissues were completely digested. Then, the remaining tissue was flushed out with water using a syringe. Females collected during the same collection event were tentatively associated to males by size and color pattern.

### ﻿Illustrations and descriptions

Pencil illustrations of the genitalia were prepared using an Olympus BX41 compound microscope with a U-DA drawing tube at 200× and 400× magnification. Additionally, an Olympus SZX12 stereo zoom microscope at 90–144× magnification was used to verify details in the illustrations. These pencil sketches were then scanned and placed into Adobe Illustrator (version CS5, Adobe Systems, Inc.) files to serve as templates and then traced to create vector graphic illustrations. A graphic tablet and pen (BAMBOO^TM^, Wacom Technology Co.) facilitated careful tracing of the original image.

**Figure 1.**
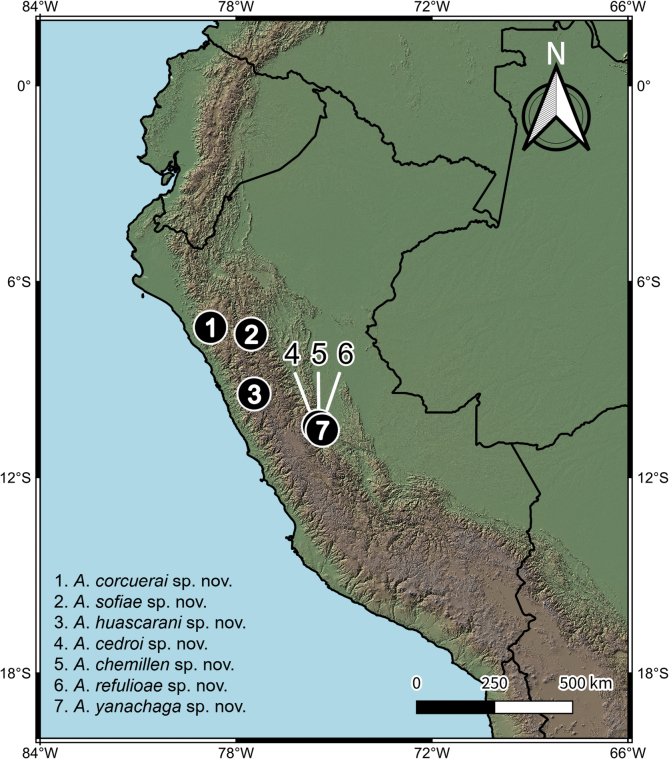
Distribution map of the new species of *Atopsyche*.

The species distribution map (Fig. 1) was prepared in QGIS 3.36.0-Maidenhead (QGIS Development Team 2024) using vector and raster data from Natural Earth (2024) and CIAT-CSI SRTM (Jarvis et al. 2008), respectively.

During our examination of the specimens included in this study, we identified two species (*A. huascarani* and *A. yanachaga*) belonging to the subgenus Dolochorema. However, the illustrations for Schmid’s species (*A. bispinosa* and *A. major*) only featured lateral views. To facilitate the identification of potential additional new species in this subgenus, we decided to borrow the type specimens for all three known species and re-illustrate them. We also present the wing venation for *A. bispinosa* and *A. irregularis*.

### ﻿Morphological terminology, descriptions, and material examined

The terminology used to describe male genitalia follows Schmid (1989), with one modification: we use the term “phallic spine” rather than “aedeagus”, as the latter is inconsistently applied by different authors to refer to the entire phallus or just its distal part (de la Torre-Bueno et al. 1989). For simplicity, paired structures are referred to in the singular.

The type specimens will be deposited in the collections of the Departamento de Entomología, Museo de Historia Natural, Universidad Nacional Mayor de San Marcos, Lima, Peru (**MUSM**); the University of Minnesota Insect Collection (**UMSP**); and the Senckenberg Research and Natural History Museum Frankfurt (**SMF**), as specified under each species treatment. Each specimen was assigned a barcode label with a unique alphanumeric sequence beginning with the UMSP prefix, serving as an exclusive identifier for specimen data uploaded to the University of Minnesota Insect Collection (UMSP) Specify database.

### ﻿Depositories

The types and materials examined for this study are deposited in the following institutions:

**CAS** California Academy of Sciences, San Francisco, California, USA

**MCZ** Museum of Comparative Zoology, Harvard University, Cambridge, Massachusetts, USA

**MUSM** Museo de Historia Natural, Universidad Nacional Mayor de San Marcos, Lima, Peru

**SMF** Senckenberg Research Institute and Natural History Museum Frankfurt, Frankfurt am Main, Germany

**USNM** National Museum of Natural History, Smithsonian Institution, Washington D.C., USA

**UMSP** University of Minnesota Insect Collection, Saint Paul, Minnesota, USA

## ﻿Results

### ﻿Species descriptions

#### ﻿ Atopsyche cedroi sp. nov.

Taxon classificationAnimaliaTrichopteraHydrobiosidae

6747C89A-47C9-592E-AA05-F961AD12D250

https://zoobank.org/A223467A-C4CD-41E1-9FEC-4D30B6CBEACA

Figs 2, 3

##### Type material.

***Holotype*.** Peru • 1♂; Pasco, Yanachaga-Chemillén NP, Quebrada San Alberto at Refugio El Cedro; 10.5452°S, 75.3578°W, 2421 m a.s.l.; 27 Aug. 2015; E. Rázuri, L. Figueroa and B. Portuguez leg.; light trap; UMSP000220106 (MUSM). ***Paratype*.** Peru • 1♀; same data as the holotype (MUSM).

##### Diagnosis.

*Atopsyche cedroi* belongs to the *bicolorata* species group of Schmid (1989), characterized by having short inferior appendages and a broad notch apically on the first segment of these appendages. The second segment is reduced and inserted at the posterodorsal corner of or in the notch of the first segment. Among the species in this group, *A. cedroi* most closely resembles *A. unicolorata* Schmid, 1989 (Bolivia). In both species, the first segment of the inferior appendage is apically notched, with the second segment inserted at the posterodorsal corner of the first segment. However, *A. cedroi* differs in several key features. The apical notch is broad, and the posteroventral corner is rectangular, while in *A. unicolorata*, the apical notch is narrower, and the posteroventral corner is digitate. Additionally, the second segment of the inferior appendage in *A. cedroi* is elongated and downturned, whereas in *A. unicolorata*, it is subtriangular and directed posterad. Although both species have a simple phallic apparatus, the base of the phallotheca is broad in *A. cedroi* and narrowly rounded in *A. unicolorata*. Finally, while the parapods are similarly shaped in both species, in *A. cedroi*, they are broader mesally and feature a pair of prominent spine-like setae on their lateral surface, which are absent in *A. unicolorata*.

##### Description.

**Figure 2.**
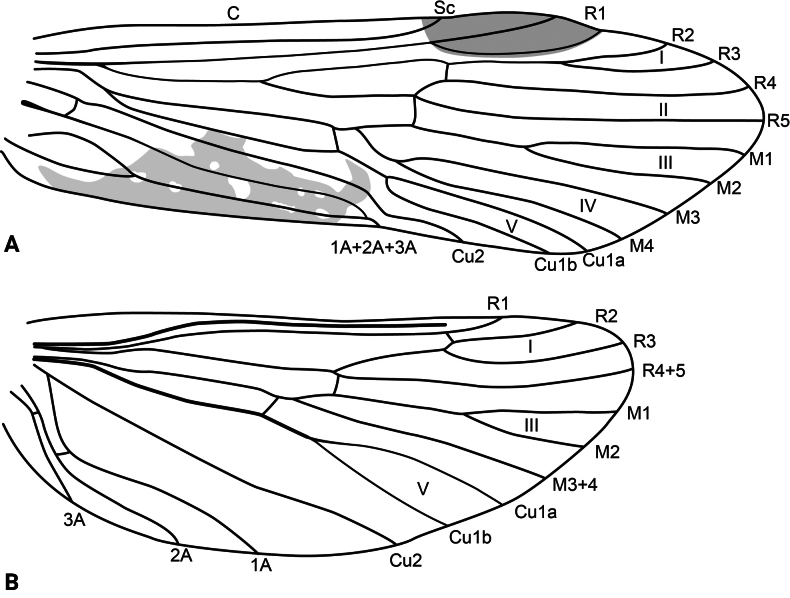
*Atopsyche cedroi* sp. nov., wing venation. **A.** Forewing; **B.** Hindwing. Abbreviations: C, costal vein; Sc, subcostal vein; R1–R5, first to fifth branches of the radial vein; R4+5, branch 4+5 of the radial vein (hindwing); M1–M4, first to fourth branches of the medial vein; M3+4, medial vein 3+4 (hindwing); Cu1a, anterior branch of first cubital vein; Cu1b, posterior branch of first cubital vein; Cu2, second cubital vein; 1A–3A, first to third anal veins; I–V, first to fifth wing forks.

**Adult.** Forewing length: male (9.5 mm, *n* = 1), female (13.5 mm, *n* = 1). Body and wings mostly pale brown, head and thorax dark brown. Forewing with scattered brown setae and longer, erect straw-colored setae along major longitudinal veins; with irregular patch of dark brown setae delimited by the posterior margin, the stem of Cu1, the apical stem of 1A+2A+3A, and the base of the wing (corresponds with darker wing membrane) and adjacent much smaller patch of black setae on fork V; pterostigma slightly coriaceous. Wing venation as in Fig. 2. Sterna III and IV without glands; sternum V with a pair of long, membranous glands; process on sternum VI slightly shorter than its segment, digitate, bearing short setae on basal third and spine-like setae on apical two-thirds; process on sternum VII very short, digitate, bare.

**Figure 3.**
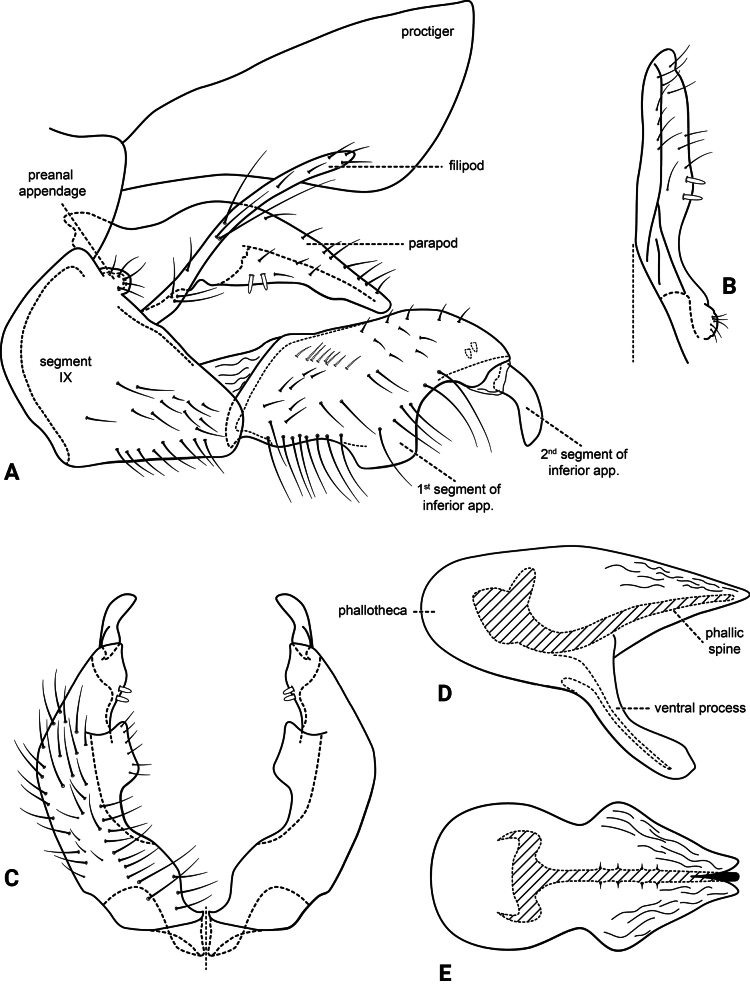
*Atopsyche cedroi* sp. nov., male genitalia. **A.** Segments IX and X, lateral; **B.** Right parapod and preanal appendage, dorsal; **C.** Inferior appendages, ventral; **D.** Phallic apparatus, lateral; **E.** Phallic apparatus, dorsal.

***Male genitalia* (Fig. 3).** Segment IX, in lateral view, subtriangular, slightly longer than high (Fig. 3A). Parapod, in lateral view, shorter than inferior appendage, broader mesally, tapering towards apex, directed slightly posteroventrad, with setae on apical half and a pair of peg-like setae on mesoventral surface, apex rounded (Fig. 3A); in dorsal view, digitate, lateral margin slightly sinuous, mesal margin straight, with setae apically, apex rounded (Fig. 3B). Filipod digitate, shorter than parapods, setose, apex acute (Fig. 3A). Preanal appendage short, rounded, setose (Fig. 3A). First segment of inferior appendage, in lateral view, roughly quadrangular, ventral margin slightly sinuous, dorsal margin slightly convex apically, posteroventral corner produced into quadrate process, posterodorsal corner produced posterad, broadly notched apically, with long setae on ventral margin, short setae on lateral surface, and pair of peg-like setae subapically on mesal surface (three peg-like setae on opposite side) (Fig. 3A); in ventral view, mitten-shaped, setose, lateral margin convex, mesal margin slightly sinuous with a quadrate process medially (Fig. 3C); second segment of inferior appendage, in lateral view, digitate, with a few very short setae basally, slightly curved posteroventrad (Fig. 3A); in ventral view, digitate, slightly curved mesad, apex narrowly rounded (Fig. 3C). Proctiger, in lateral view, broadly widened apically, with truncate apical margin, covered with small setae (visible at 100×) (Fig. 3A); in dorsal view, tapering apically. Phallic apparatus simple; phallotheca broadly rounded basally, phallic apodeme indiscernible; with ventral process articulating with inferior appendages, broad, constricted mesally; ventrolateral branches of phallotheca absent; dorsal process of phallotheca absent; posterior section of the phallotheca, in lateral view, broad basally, tapering towards apex, directed posterad, apex acute, membranous apically (Fig. 3D); in dorsal view, with a notch mesally, apex directed posterad (Fig. 3E); phallic spine elongate, stout, with a slight curvature near the base (Fig. 3D); in dorsal view, with four erect setae on small, lateral bulges, apex acute (Fig. 3E).

##### Distribution.

Peru: Pasco Department.

##### Etymology.

This new species is named after the locality where the type was collected, Refugio El Cedro (Yanachaga-Chemillén NP, Pasco Department, Peru).

#### ﻿ Atopsyche chemillen sp. nov.

Taxon classificationAnimaliaTrichopteraHydrobiosidae

76787BDA-787E-5434-A9A9-7C78AAAE9CB4

https://zoobank.org/DFAD726B-545F-4229-BA94-67BED254DD9E

Figs 4, 5

##### Type material.

***Holotype*.** Peru • 1♂; Pasco, small creek in Yanachaga-Chemillén NP buffer zone, San Daniel sector; 10.4278°S, 75.4732°W, 2134 m a.s.l.; 03 May 2011; C. Carranza, M. Alvarado, L. Figueroa leg.; light trap; MUSM-ENT-320759 (MUSM). ***Paratypes*.** Peru • 3♂; same data as the holotype, but 04 May 2011 • 1♂; same data as the holotype, but 16 Jun. 2010; E. Rázuri, C. Carranza leg. (MUSM) • 1♂; same data as the holotype, but 16 Nov. 2010; C. Carranza, J. Peralta leg. (MUSM) • 1♂ 1♀; same data as the holotype, but 24 Aug. 2015; E. Rázuri, L. Figueroa, B. Portuguez leg. (UMSP) • 2♀; same data as the holotype, but 24 Aug. 2015; E. Rázuri, L. Figueroa, B. Portuguez leg. (MUSM).

##### Diagnosis.

*Atopsyche chemillen* is most similar to members of the *falina* species group of Ross and King (1952). This species group is characterized by the slightly produced posteroventral corner of the first segment of the inferior appendage. From all the members included therein, the new species is most similar to *A. falina* (Navás, 1930) (Argentina), *A. mayucapac* Schmid, 1989 (Venezuela), *A. neolobosa* Flint, 1963 (Ecuador), and *A. yunguensis* Rueda Martín, 2006 (Argentina, Bolivia) based on the presence of a spine-bearing dorsal process on the phallotheca. *Atopsyche chemillen* is most similar to *A. mayucapac* based on the straight inferior appendage and the shape of the phallotheca, but differs in the shape of the parapods in lateral view (the dorsal margin of this structure is biconcave in the new species but almost straight in *A. mayucapac*) and the shape of segment IX (quadrate in the new species but the dorsal margin is obliterated in *A. mayucapac*).

##### Description.

**Figure 4.**
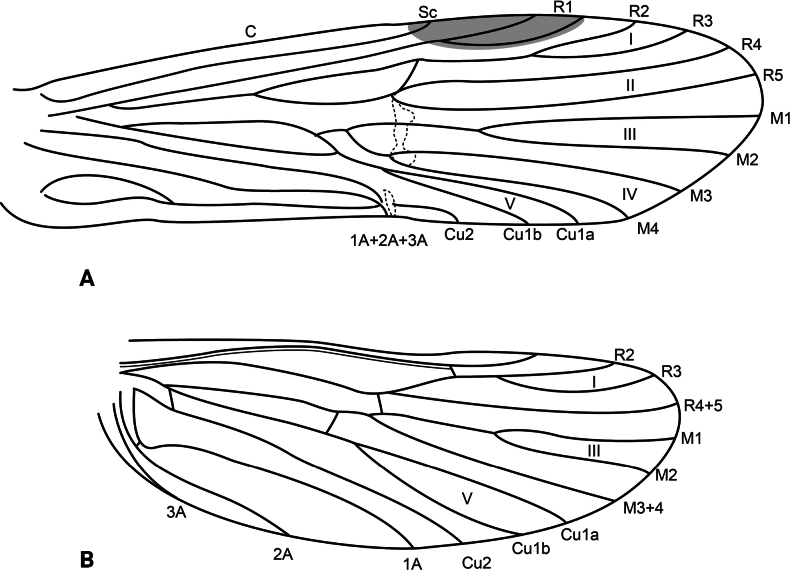
*Atopsyche chemillen* sp. nov., wing venation. **A.** Forewing; **B.** Hindwing.

**Adult.** Forewing length: male (9 mm, *n* = 1), female (11 mm, *n* = 1). Body pale brown, wings brown. Forewing with scattered dark brown setae and longer straw-colored and brown setae along major longitudinal veins and wing margin. Wing venation as in Fig. 4. Tergum IV with elongate, flattened gland, opening on posterolateral margin, lined internally with minute spines; sternum V with a pair of tiny protuberances; process on sternum VI as long as its segment, curved, bearing spine-like setae along its length (the last one is peg-like), process on sternum VII shorter than process on previous segment, digitate, bearing very fine, short setae (only visible at 200×).

**Figure 5.**
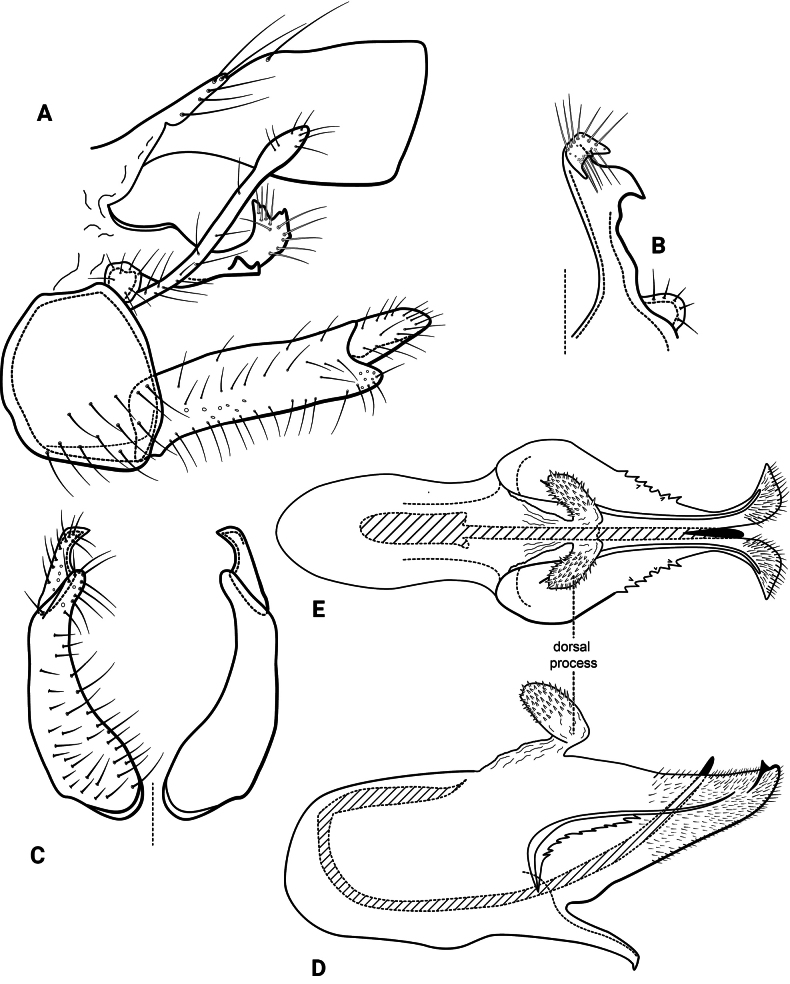
*Atopsyche chemillen* sp. nov., male genitalia. **A.** Segments IX and X, lateral; **B.** Right parapod and preanal appendage, dorsal; **C.** Inferior appendages, ventral; **D.** Phallic apparatus, lateral; **E.** Phallic apparatus, dorsal.

***Male genitalia* (Fig. 5).** Segment IX, in lateral view, quadrangular, slightly higher than long (Fig. 5A). Parapod, in lateral view, shorter than inferior appendage, same width along its length, with a spine-like lobe subapically on ventral margin, directed posterodorsad, with setae apically, apex concave (Fig. 5A); in dorsal view, short, lateral margin with a spine-like projection subapically, mesal margin straight, with setae apically, apex upturned, concave (Fig. 5B). Filipod digitate, longer than parapods, setose, apex slightly capitate (Fig. 5A). Preanal appendage short, rounded, setose (Fig. 5A). First segment of inferior appendage, in lateral view, long, straight, same width along its length, posteroventral corner slightly produced posterad, with setae on lateral and ventral surfaces (Fig. 5A); in ventral view, slightly C-shaped, setose, lateral margin almost straight, mesal margin slightly concave, posterior margin truncate (Fig. 5C); second segment of inferior appendage, in lateral view, subtriangular, very short, setose, dorsal and ventral margins straight, apex rounded (Fig. 5A); in ventral view, falcate, apex acute (Fig. 5C). Proctiger, in lateral view, broadly widened apically, with truncate apical margin, with a few long setae on its basal half; in dorsal view, tapering apically (Fig. 5A). Phallic apparatus complex; phallotheca broadly rounded basally, phallic apodeme indiscernible; with ventral process articulating with inferior appendages, narrow, tapering towards apex; ventrolateral branches of phallotheca absent; dorsal process of phallotheca present, divided into pair of recurved, rounded lobes, bearing short spines on apical half; posterior section of phallotheca, in lateral view, broad basally, tapering towards apex, directed posterad, apical half covered with short setae, lateral surface with a serrated ridge, apex narrowly rounded (Fig. 5D); in dorsal view, with a deep notch mesally, apex directed laterad (Fig. 5E); phallic spine elongate, stout, with a strong curvature near the base (Fig. 5D); in dorsal view, apex acute (Fig. 5E).

##### Distribution.

Peru: Pasco Department.

##### Etymology.

*Atopsyche chemillen* is named after the Yanachaga-Chemillén National Park, where the type locality is situated. *Chemillen* means black or burned in Yanesha, referring to the dark appearance of these mountains during sunsets on sunny days.

#### ﻿ Atopsyche corcuerai sp. nov.

Taxon classificationAnimaliaTrichopteraHydrobiosidae

F5F70BF4-D001-5CDD-8CF8-1F7B000625A8

https://zoobank.org/B0582326-A7FE-4727-86BE-5F0FE03B9A04

Figs 6, 7

##### Type material.

***Holotype*.** Peru • 1♂; Cajamarca; ACP Bosque Cachil; 7.39689°S, 78.7809°W, 2522 m a.s.l.; 21–22 Apr. 2015; J. Peralta, P. Sánchez, M. Rodríguez, E. Sánchez, D. Silva, J. Grados leg.; light trap; MUSM-ENT-320967 (MUSM). ***Paratypes*.** Peru • 9♂ 1♀; same data as the holotype (MUSM) • 2♂; same data as the holotype (UMSP) • 2♂; same data as the holotype (NMNH) • 2♂; same data as the holotype (SMF).

##### Diagnosis.

This new species closely resembles *Atopsyche cajas* Harper & Turcotte, 1985 (Ecuador), particularly in the position of the second segment of the inferior appendage, which is inserted on the mesal surface of the first segment. However, the two species can easily be separated based on differences in the shape of the parapod, inferior appendage, and phallic apparatus. In the new species, the parapod is narrow and tapering in lateral view, while in *A. cajas*, the parapod is broader and roughly uniform in width. In dorsal view, the parapod of the *A. corcuerai* is narrow and truncate apically, whereas in *A. cajas*, it is wider mesally and rounded apically. The apex of the first segment of the inferior appendages in *A. corcuerai* is falcate, while in *A. cajas*, it is broadly rounded. In ventral view, the second segment of the inferior appendages in *A. corcuerai* is roughly one-third the length of the first segment, while in *A. cajas*, it is about half the length of the first segment. Additionally, the mesal surface of the first segment of the inferior appendage is produced in *A. corcuerai*, whereas it is rounded in *A. cajas*. Finally, the posterior processes of the phallotheca in the new species are longer and narrower than those in *A. cajas*, and the phallic spine is straight in the new species, but slightly curved in *A. cajas*.

##### Description.

**Figure 6.**
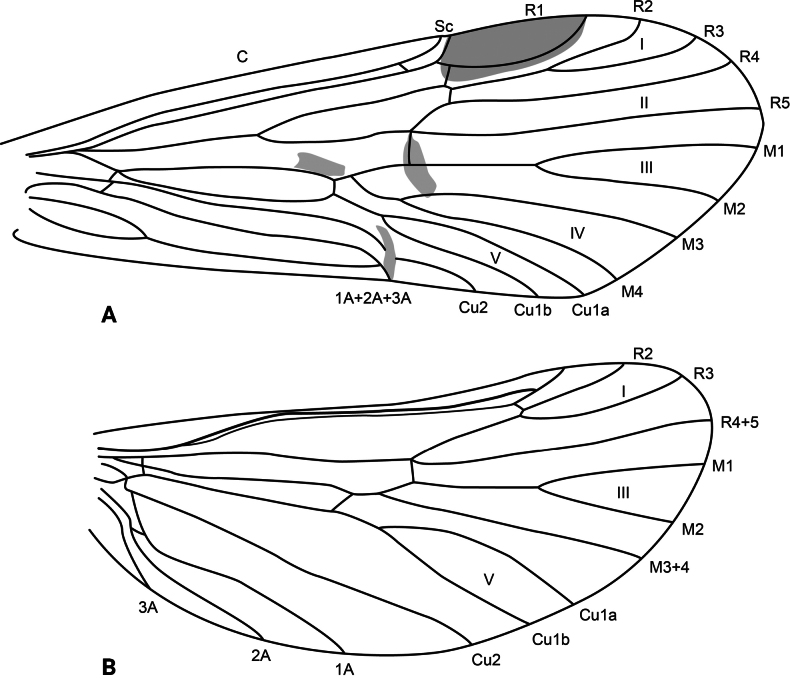
*Atopsyche corcuerai* sp. nov., wing venation. **A.** Forewing; **B.** Hindwing.

**Adult.** Forewing length: male (9 mm, *n* = 16), female (10 mm, *n* = 1). Body brown (in ethanol), wings pale brown (in ethanol). Wing venation as in Fig. 6. Sternum III–IV without glands; sternum V with a pair of long, membranous glands; process on sternum VI slightly longer than its segment, slightly curved, bearing fine setae on its basal half and spine-like setae on its apical half, process on sternum VII very short, subtriangular, bearing very fine setae.

**Figure 7.**
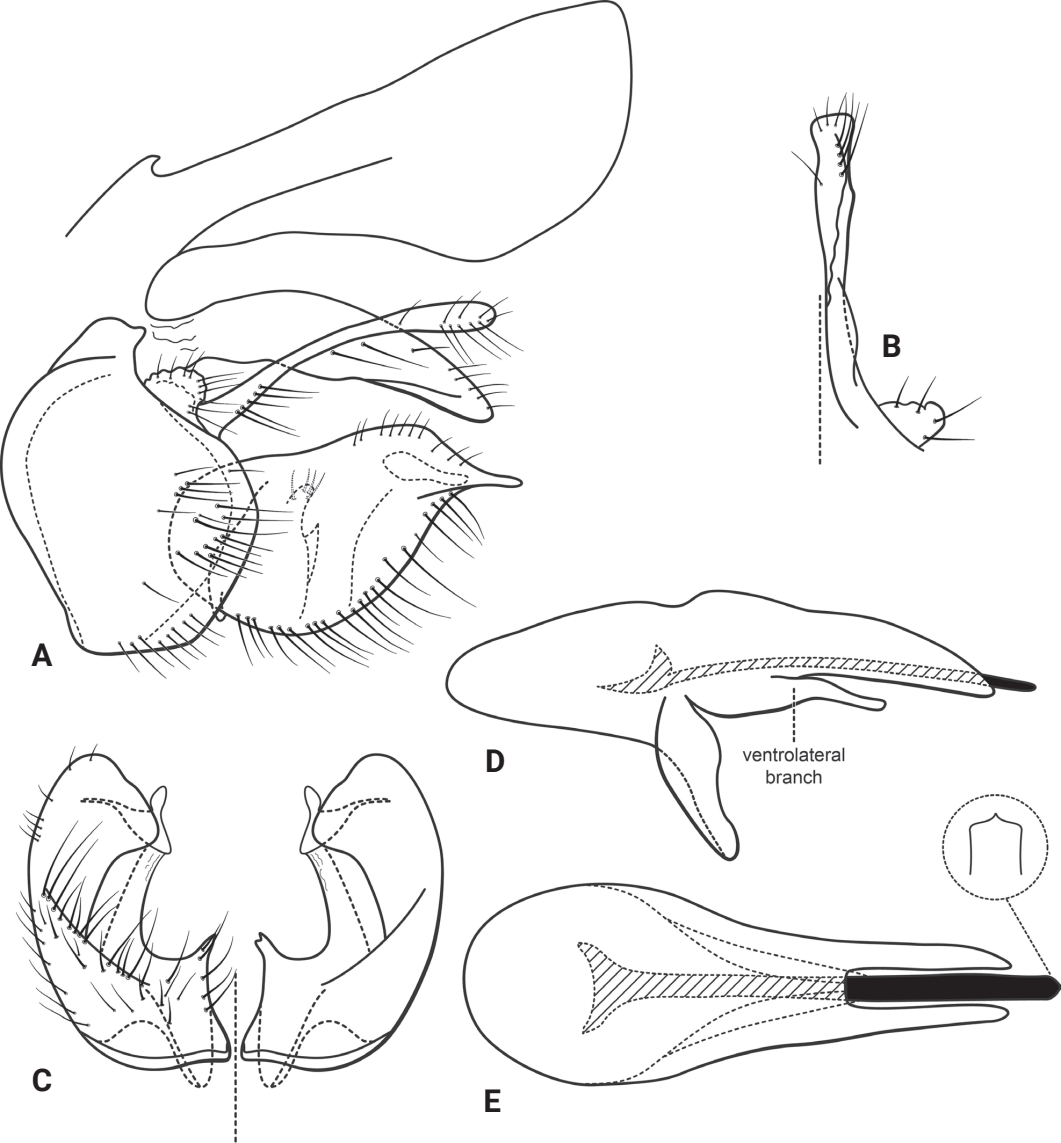
*Atopsyche corcuerai* sp. nov., male genitalia. **A.** Segments IX and X, lateral; **B.** Right parapod and preanal appendage, dorsal; **C.** Inferior appendages, ventral; **D.** Phallic apparatus, lateral; **E.** Phallic apparatus, dorsal.

***Male genitalia* (Fig. 7).** Segment IX, in lateral view, pentagonal, shorter than high, with setae on posterolateral and posteroventral surface (Fig. 7A). Parapod, in lateral view, elongate, sausage-shaped, broad mesally, tapering towards apex, directed slightly ventrad, dorsal and mesal surface with a few setae subapically, apex narrowly rounded (Fig. 7A); in dorsal view, elongate, lateral margin slightly sinuous, mesal margin almost straight, setae on dorsal surface on apical half, apex truncate (Fig. 7B). Filipod digitate, as long as parapods, setose, apex rounded (Fig. 7A). Preanal appendage short, rounded, setose (Fig. 7A). First segment of inferior appendage, in lateral view, oval, ventral margin convex, dorsal margin slightly convex subapically, posterior margin produced into short, subacute process, with setae along on ventral margin and dorsal surface subapically (Fig. 7A); in ventral view, reniform, setose, lateral margin convex, mesal margin concave and slightly membranous with a mesal projection directed posteromesad (this projection is pointed on the right side and presents a slight cleft on the left side) (Fig. 7C); second segment of inferior appendage, in lateral view, less than half the length of the first segment, ovate, dorsal and ventral margins slightly constricted mesally, apex subacute (Fig. 7A); in ventral view, subtriangular, posterior margin slightly projected posterad, apex narrowly rounded (Fig. 7C). Proctiger, in lateral view, narrow basally, wider apically, with a short carina laterodorsally on basal half, covered with minute setae, apex slightly rounded (Fig. 7A); in dorsal view, tapering apically, with a slight mesal notch. Phallic apparatus complex; phallotheca broadly rounded basally, phallic apodeme indiscernible; with ventral process articulating with inferior appendages, broad, slightly broader basally; ventrolateral branches of phallotheca present, roughly digitate, approximately 0.66 times as long as posterior half of phallotheca, blunt apically; dorsal process of phallotheca absent; posterior section of phallotheca, in lateral view, broad basally, tapering towards apex, directed posterad, apex narrowly rounded (Fig. 7D); in dorsal view, with a deep notch mesally (Fig. 7E); phallic spine elongate, stout, spine-like structure, without basal curvature, straight (Fig. 7D); in dorsal view, slightly pointed apically (Fig. 7E).

##### Distribution.

Peru: Cajamarca Department.

##### Etymology.

This new species is named in honor of the Peruvian poet Marco Antonio Corcuera Díaz, who heralded the protection of Bosques de Cachil, where the type series was collected.

#### ﻿ Atopsyche (Dolochorema) huascarani sp. nov.

Taxon classificationAnimaliaTrichopteraHydrobiosidae

6E4E3262-C786-5B35-AE57-A51959387962

https://zoobank.org/EC19490D-9951-4239-93A0-AD542E4FA91E

Figs 8, 9

##### Type material.

***Holotype*.** Peru • 1♂; Ancash, Huascarán NP, Puesto de control Llaca, Laguna Llaca; 9.4375°S, 77.4481°W, 4421 m a.s.l.; 09 May 2016; P. Ancajima and R. Pradel leg.; light trap; MUSM-ENT-320969 (MUSM). ***Paratypes*.** Peru • 1♂; same data as the holotype (UMSP) • 1♀; same data as the holotype (MUSM).

##### Diagnosis.

*Atopsyche yanachaga* belongs to a group of species traditionally included in the subgenus Dolochorema. These species are characterized by the insertion of the second segment of the inferior appendage, which is positioned on the mesal surface of the first segment and is largely hidden in lateral view. Among these species, *A. yanachaga* is most similar to *A. irregularis* (Banks, 1913) (Peru) and *A. bispinosa* Schmid, 1989 (Bolivia) based on the shape of the parapods in lateral and dorsal view and the inferior appendages in ventral view. All three species have an elongate, narrow parapod, but both *A. yanachaga* and *A. irregularis* possess a strongly bent parapod at the base. In contrast, in dorsal view, *A. yanachaga* has an inflated, rounded parapod apically, while *A. irregularis* has a straight, narrow one. The first segment of the inferior appendages in these species is mitten-shaped in ventral view, but they can be distinguished by the shape of the second segment. In *A. yanachaga*, this segment extends beyond the posterior margin of the first segment, while in the other two species, they are small. Notably, *A. yanachaga* is the only species in this group that lacks the ventrolateral branch of the phallic apparatus.

##### Description.

**Figure 8.**
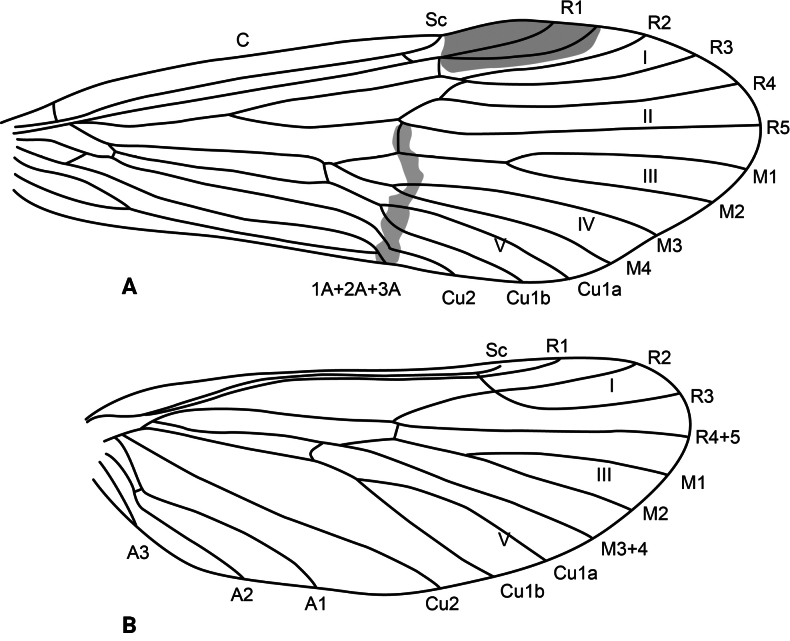
*Atopsyche huascarani* sp. nov., wing venation. **A.** Forewing; **B.** Hindwing.

**Adult.** Forewing length: male (12.5 mm, *n* = 2), female (13.5 mm, *n* = 1). Body brown (in alcohol), wings pale brown (in alcohol). Wing venation as in Fig. 8. Sterna III and IV without glands; sternum V with a pair of long, membranous glands; processes on sterna VI and VII short, triangular in ventral view, setose.

**Figure 9.**
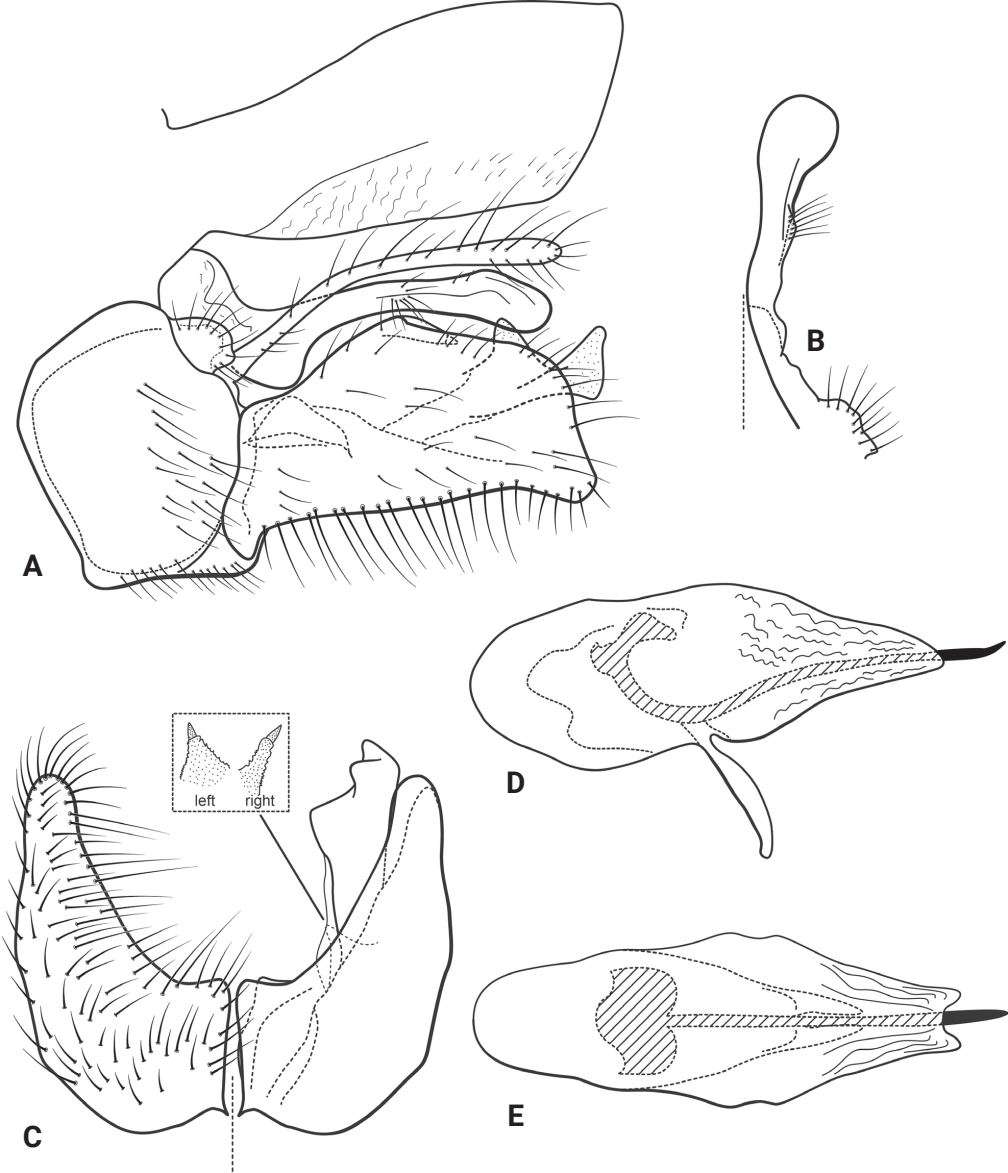
*Atopsyche huascarani* sp. nov., male genitalia. **A.** Segments IX and X, lateral; **B.** Right parapod and preanal appendage, dorsal; **C.** Inferior appendages, ventral (inset: left and right spine-bearing processes on mesodorsal surface); **D.** Phallic apparatus, lateral; **E.** Phallic apparatus, dorsal.

***Male genitalia* (Fig. 9).** Segment IX, in lateral view, pentagonal, slightly higher than long, with setae on ventral and posterolateral surface (Fig. 9A). Parapod, in lateral view, elongate, narrow, broader and strongly bent basally, apex rounded, dorsal margin and lateral surface with a few setae mesally (Fig. 9A); in dorsal view, elongate, lateral margin sinuous, mesal margin convex mesally, setae on dorsal surface at mid-length, apex inflated and rounded (Fig. 9B). Filipod digitate, as long as parapods, setose, apex rounded (Fig. 9A). Preanal appendage short, rounded, setose (Fig. 9A). First segment of inferior appendage, in lateral view, rectangular, ventral margin straight, slightly expanded basally and apically (less produced in the paratype), dorsal margin convex at mid-length, produced mesad into a rugose, triangular projection, bearing single spine-like setae apically (less slender in one of the sides of the paratype), with setae along margins and lateral surface (Fig. 9A); in ventral view, mitten-shape, setose, lateral margin convex, mesal margin concave (Fig. 9C); second segment of inferior appendage, in lateral view, elongate, bilobed, dorsal lobe subacute and directed dorsad, ventral lobe triangular, inserted on the mesal surface of the first segment of the inferior appendage (Fig. 9A); in ventral view, roughly quadrangular, apicolateral corner produced into a rounded lobe, apex truncate (Fig. 9C). Proctiger, in lateral view, narrow basally, slightly wider apically, with a short carina basolaterally, ventral margin membranous, densely covered with very fine setae, apex truncate (Fig. 9A). Phallic apparatus simple; phallotheca broadly rounded basally, phallic apodeme indiscernible; with narrow ventral process articulating with inferior appendages, slightly broader at mid-length; ventrolateral branches of phallotheca absent; dorsal process of phallotheca absent; posterior section of phallotheca, in ventral view, broad basally, tapering towards apex, directed posterad, apex subacute (Fig. 9D); in dorsal view, with a shallow notch mesally (Fig. 9E); phallic spine, stout, spine-like structure, with a strong convex curvature near base, then slightly sinuous (Fig. 9D); in dorsal view, apex acute (Fig. 9E).

##### Distribution.

Peru: Ancash Department.

##### Etymology.

The specific epithet refers to the Huascarán National Park, from where the type specimen was collected.

#### ﻿ Atopsyche refulioae sp. nov.

Taxon classificationAnimaliaTrichopteraHydrobiosidae

0EDB7B8C-7261-5FB9-9824-A246DB506B00

https://zoobank.org/4C020E8D-69E5-44B6-BC96-51C7A48985D8

Figs 10, 11

##### Type material.

***Holotype*.** Peru • 1♂; Pasco, Yanachaga-Chemillén NP, Quebrada San Alberto at Refugio El Cedro; 10.5452°S, 75.3578°W, 2421 m a.s.l.; 27 Aug. 2015; E. Rázuri, L. Figueroa and B. Portuguez leg.; light trap; UMSP000220104 (MUSM). ***Paratypes*.** Peru • 1♂ 2♀; same data as the holotype (MUSM) • 1♂ 2♀; same data as the holotype (UMSP).

##### Diagnosis.

*Atopsyche refulioae* is related to species included in the *A. batesi* species group due to the unpaired dorsobasal process of the phallic apparatus. Among these species, *A. refulioae* is most similar to *A. callosa* Navás, 1924 (Colombia, Costa Rica, Ecuador, Panama, Peru, Venezuela), *A. majada* Ross, 1947 (Belize, Costa Rica, Guatemala, Honduras, Nicaragua, Mexico, Panama), *A. minimajada* Blahnik & Gottschalk, 1997 (Costa Rica), and *A. puharcocha* Schmid, 1989 (Bolivia, Ecuador, Peru) based on the shape of segment IX in lateral view and the presence of dorsal process on the phallic apparatus. *Atopsyche refulioae* can easily be distinguished from these species based on the shape of the first segment of the inferior appendage with the posteroventral corner produced into a point, which is produced into a digitate process of varying length in the other species. Also, the claw-like shape of the parapod in dorsal view (elongate and sinuous in lateral view) sets the new species apart from the other species, which have shorter and wider parapods.

##### Description.

**Figure 10.**
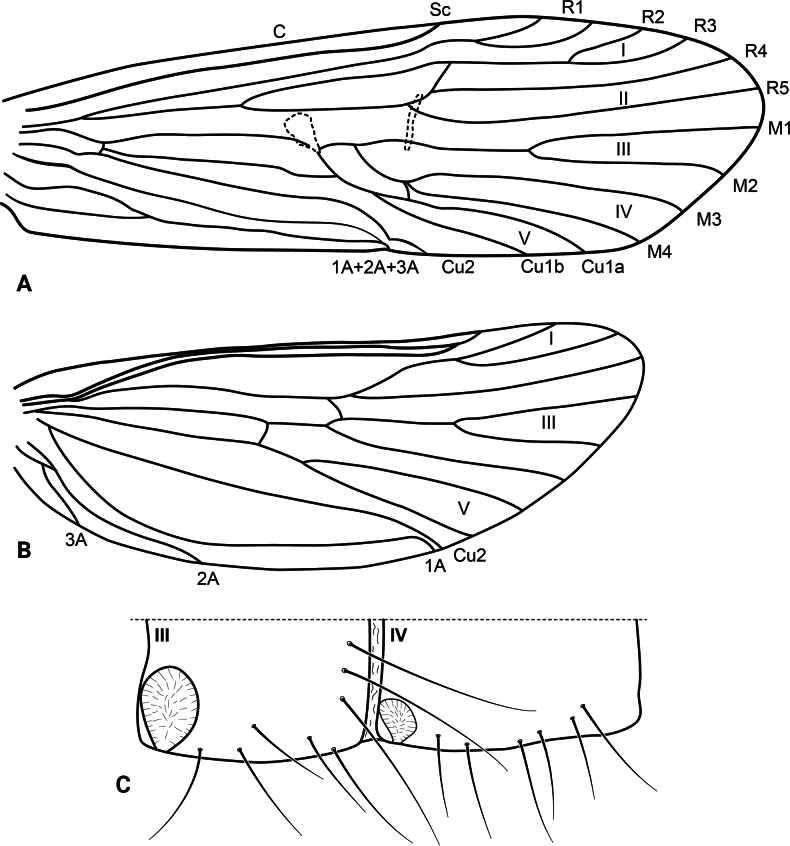
*Atopsyche refulioae* sp. nov., wing venation. **A.** Forewing; **B.** Hindwing; **C.** Abdominal glands on terga III and IV.

**Adult.** Forewing length: male (7.25 mm, *n* = 2). Body pale brown, wings dark brown in the area delimited by the costal vein, the bifurcation of the R1, the base of the M vein, and the vein that closes the discal cell, and along the apical margin, otherwise pale brown. Forewing with erect setae on veins forming irregular pattern of alternate dark brown and yellow setae, with dark brown setae along costal margin; apex of wing with fringe of brown and yellow setae. Wing venation as in Fig. 10A, B. Terga III and IV with oval glands, lined internally with spines (glands on tergum IV are smaller) (Fig. 10C); process on sternum VI longer than its segment, slightly curved, bearing fine setae on its basal half increasing in thickness towards its apex, last few setae very prominent and spine-like; process on sternum VII short, less than half the length of its segment, straight, bare.

**Figure 11.**
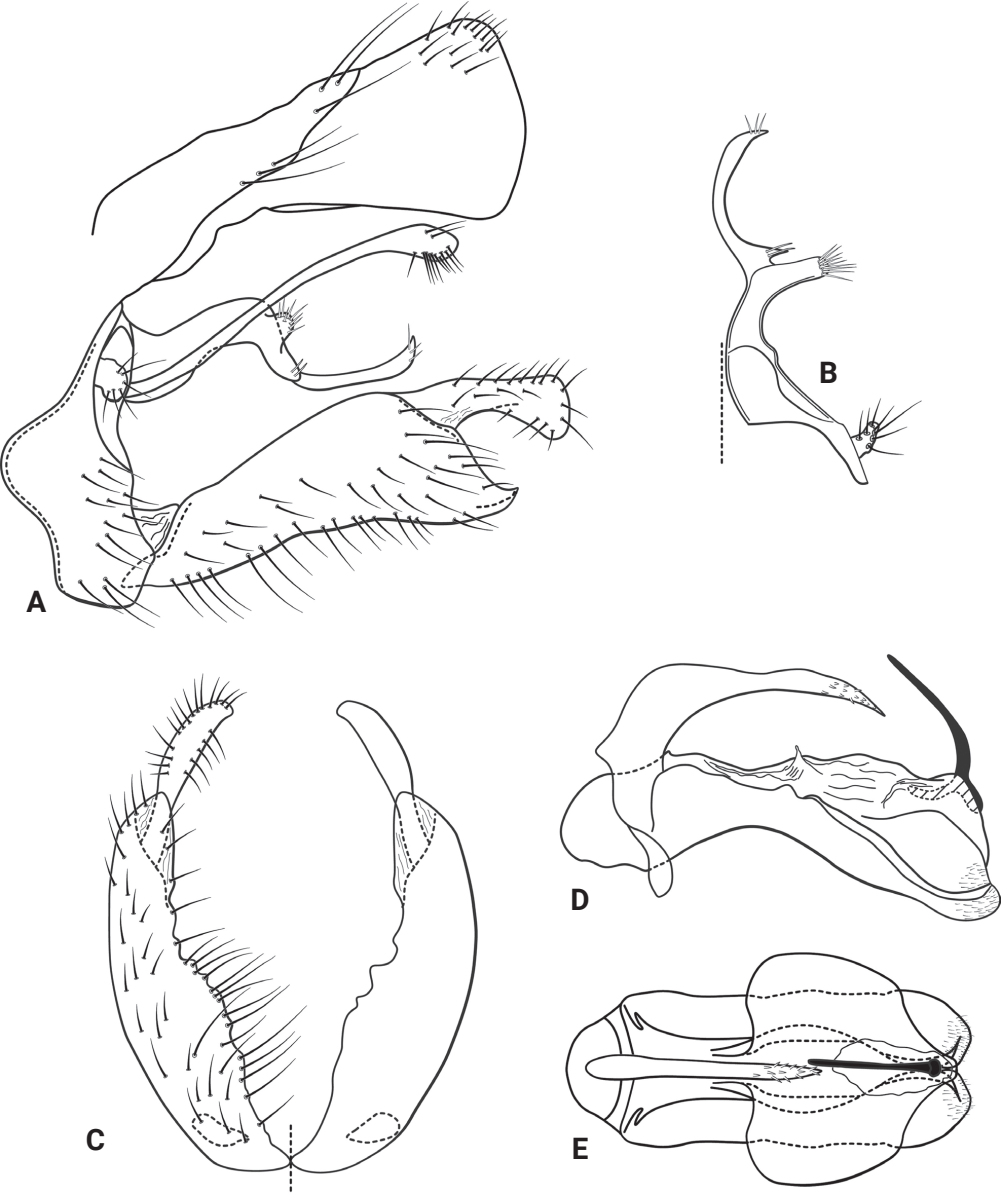
*Atopsyche refulioae* sp. nov., male genitalia. **A.** Segments IX and X, lateral; **B.** Right parapod and preanal appendage, dorsal; **C.** Inferior appendages, ventral; **D.** Phallic apparatus, lateral; **E.** Phallic apparatus, dorsal.

***Male genitalia* (Fig. 11).** Segment IX, in lateral view, quadrangular, much higher than long, dorsal margin very short, with setae on posterolateral and ventral surfaces (Fig. 11A). Parapod in lateral view, broad basally, narrow apically, sinuous, mesally with a spinose acute process and a dorsal, rounded projection bearing long setae, apex acute and upturned, bearing three long setae (Fig. 11A); in dorsal view, broad basally with transverse carina, mesal margin concave and lateral margin biconcave, mesal rounded projection, spinose acute process, and acute apex directed laterad (Fig. 11B). Parapod, in lateral view, elongate, sinuous, broader basally, tapering into an acute, upturned apex, mesally with a spinose subacute process and a dorsal rounded projection bearing long setae, with short setae apically (Fig. 11A); in dorsal view, elongate, sinuous, mesal processes directed laterad, most basal process rounded and bearing long setae, process at mid-length acute and bearing spine-like setae, acute apically. Filipod digitate, longer than parapods, with a patch of setae apically (Fig. 11A). Preanal appendage short, rounded, setose (Fig. 11A). First segment of inferior appendage, in lateral view, trapezoidal, ventral margin slightly concave, dorsal margin slightly convex subapically, posteroventral corner produced into a slightly upturned point, with setae on lateral surface and along ventral margin (Fig. 11A); in ventral view, C-shaped, setose, lateral margin convex, mesal margin sinuous (this margin differs between the left and right sides) (Fig. 11C); second segment of inferior appendage, in lateral view, finger-like, narrow at mid-length, slightly inflated apically, setose, dorsal margin convex, ventral margin concave, apex truncate (Fig. 11A); in ventral view, digitate, slightly curved mesad, apex rounded (Fig. 11C). Proctiger, in lateral view, narrow basally, wider apically, with a carina laterodorsally, ventral margin membranous basally, with long setae on apicodorsal corner and along carina, apex truncate (Fig. 11A). Phallic apparatus complex; phallotheca broadly rounded basally, phallic apodeme indiscernible; with ventral process of the phallotheca articulating with inferior appendages; ventrolateral branches of the phallotheca absent, dorsal process of the phallotheca present, single, elongate, bearing short spines apically, apex acute, with ventrolateral flanges fused to ventral process of the phallotheca; posterior section of the phallotheca, in lateral view, same width throughout its length, directed slightly posteroventrad, apex covered with short setae, lateral surface produced into a large flap, produced laterad, apex rounded (Fig. 11D); in dorsal view, with a narrow notch mesally, apex directed posterad (Fig. 11E); phallic spine elongate, stout, with a slight ventral curvature near base, then straight (Fig. 11D); in dorsal view, apex acute (Fig. 11E).

##### Distribution.

Peru: Pasco Department.

##### Etymology.

The first author would like to dedicate this new species to Sonia Refulio for her support throughout the years.

#### ﻿ Atopsyche sofiae sp. nov.

Taxon classificationAnimaliaTrichopteraHydrobiosidae

50F5A396-B209-5350-80E5-6DEA8BCE81BB

https://zoobank.org/28907D37-47EA-43FF-BD31-82E01822DFD0

Figs 12, 13

##### Type material.

***Holotype*.** Peru • 1♂; La Libertad, Provincia Bolivar, Laguna Quishuar; 7.6049°S, 77.5376°W, 3482 m a.s.l.; 31 Mar. 2011; C. Carranza leg.; light trap; MUSM-ENT-320966 (MUSM). ***Paratype*.** Peru • 1♂; same data as the holotype (UMSP).

##### Diagnosis.

*Atopsyche sofiae* also belongs to the *bicolorata* species group of Schmid (1989), characterized by having short inferior appendages and a broad notch apically on the first segment of these appendages. The second segment is reduced and inserted at the posterodorsal corner or in the notch of the first segment. Among the species in this group, the new species most closely resembles *A. yupanqui* Schmid, 1989 (Venezuela). In both species, the first segment of the inferior appendage is rectangular, with the posterodorsal and posteroventral corners slightly projecting mesad rather than posterad, and the second segment is inserted in the notch formed by these corners. In *A. sofiae*, however, these corners are more pronounced, forming a concavity where the strongly reduced second segment is inserted (not visible in lateral view). Additionally, the posterodorsal corner is densely covered with peg-like setae and the posteroventral corner bears thick, spine-like setae (features absent in *A. yupanqui*). Though both species have a quite simple phallic apparatus, the ventrolateral branch of the phallotheca in *A. sofiae* curves ventrad, while in *A. yupanqui* it is directed posterad. Finally, the parapods are slender in both species, but in *A. sofiae*, the parapods taper to an acute apex and bear a pair a peg-like setae basally. In contrast, the parapods in *A. yupanqui* do not taper, have a notched apex, and bear no peg-like setae.

##### Description.

**Figure 12.**
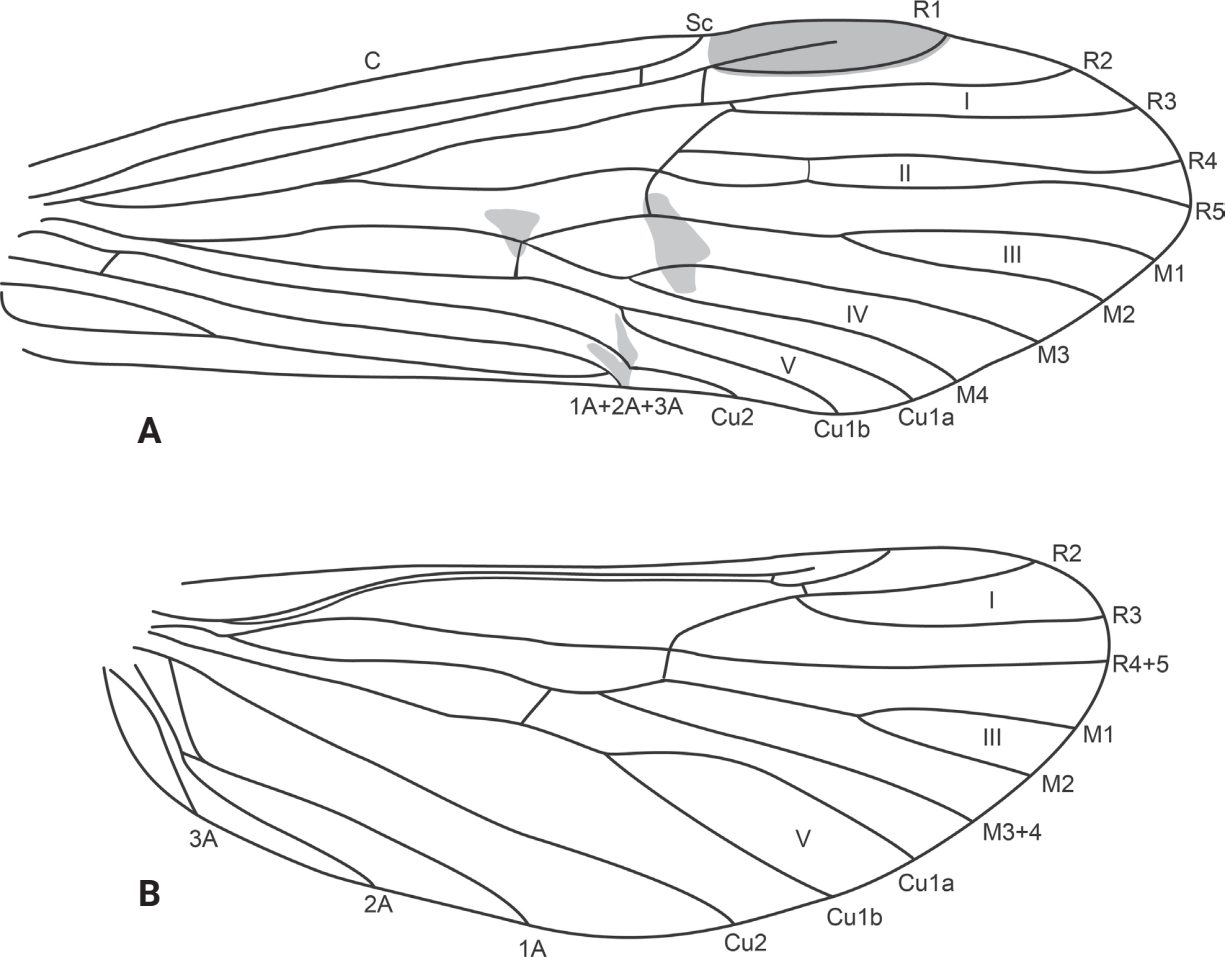
*Atopsyche sofiae* sp. nov., wing venation. **A.** Forewing; **B.** Hindwing.

**Adult**. Forewing length: male (13.5 mm, *n* = 2). Body and wings pale brown. Forewings with erect setae on veins without distinct pattern, with dark brown setae along costal margin, wing apex with fringe of brown setae. Wing venation as in Fig. 12. Sternum III and IV without glands; sternum V with a pair of long, membranous glands; processes on sternum VI and VII strongly reduced; process on sternum VI rounded in ventral view, with four spine-like setae, process on sternum VI triangular in ventral view, with densely clumped group of 8 spine-like setae with blunt apices (this process is absent in the paratype).

**Figure 13.**
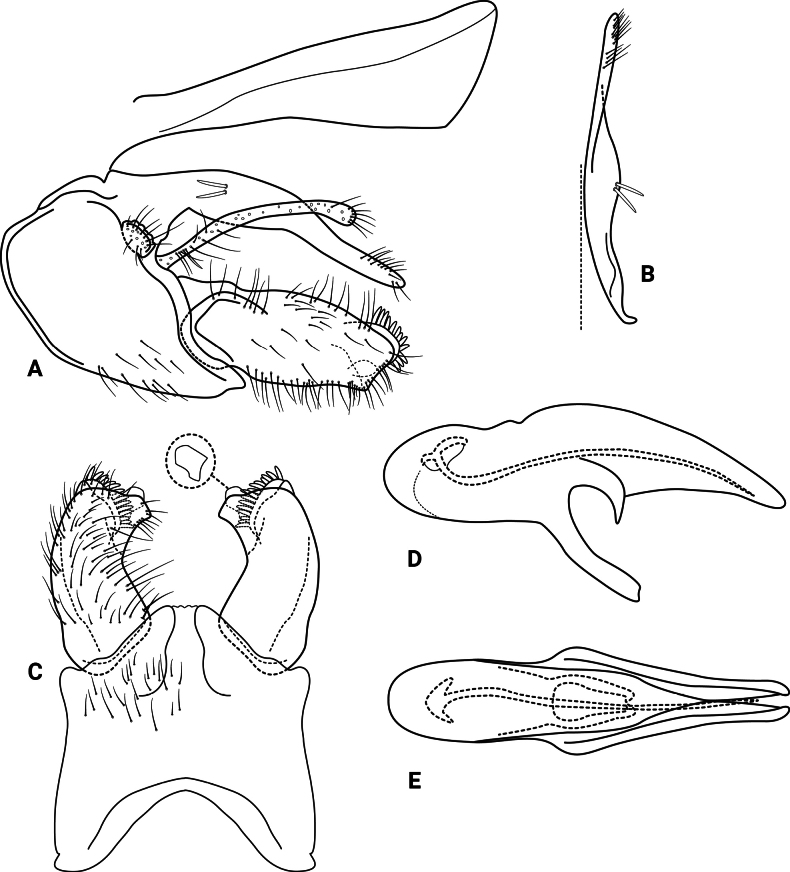
*Atopsyche sofiae* sp. nov., male genitalia. **A.** Segments IX and X, lateral; **B.** Right parapod and preanal appendage, dorsal; **C.** Inferior appendages and segment IX, ventral (inset: second segment of inferior appendage); **D.** Phallic apparatus, lateral; **E.** Phallic apparatus, dorsal.

***Male genitalia* (Fig. 13).** Segment IX, in lateral view, quadrangular, almost as high as long, posteroventral margin produced and blunt (Fig. 13A). Parapod, in lateral view, elongate, digitate, slightly broader basally, tapering towards apex, directed slightly ventrad, with a group of setae apically and pair of peg-like setae basolaterally, apex narrowly rounded (Fig. 13A); in dorsal view, flattened, lateral margin slightly sinuous, mesal margin almost straight, with a group of setae apically and peg-like setae on lateral flange, apex acute (Fig. 13B). Filipod digitate, longer than parapods, setose (Fig. 13A). Preanal appendage short, rounded, setose (Fig. 13A). First segment of inferior appendage, in lateral view, rectangular, ventral margin almost straight, slightly concave basally (left side on paratype much more convex), dorsal margin slightly convex mesally, posteroventral and posterodorsal corner produced mesad forming a socket where the second segment is inserted (left posteroventral corner on paratype directed more ventromesad), posterodorsal corner densely covered with short, peg-like setae, posteroventral corner with long, spine-like setae, with setae on dorsal, ventral, and apical margins and lateral surface (Fig. 13A); in ventral view, reniform, setose, lateral margin slightly convex, mesal margin convex with apical truncate projection (Fig. 13C); second segment of inferior appendage, in lateral view, reduced, rounded (Fig. 13A); in ventral view, reniform, slightly curved mesad, apex truncate (Fig. 13C). Proctiger, in lateral view, narrow basally, slightly wider apically, with a long diagonal carina, slightly membranous, apex truncate (Fig. 13A). Phallic apparatus complex; phallotheca broadly rounded basally, phallic apodeme indiscernible; with ventral process articulating with inferior appendages, narrow, same width throughout its length; ventrolateral branches of phallotheca present, hooked, ~ 0.25× as long as posterior half of phallotheca, curved ventrad, acute apically; dorsal process of phallotheca absent; posterior section of phallotheca, in lateral view, broad basally, tapering towards apex, with a faint ridge on lateral margin, directed slightly posteroventrad, apex narrowly rounded (Fig. 13D); in dorsal view, with a deep notch mesally (Fig. 13E); phallic spine elongate, stout, spine-like structure, with slight curvature near base, then slightly sinuous (Fig. 13D); in dorsal view, apex acute (Fig. 13E).

##### Distribution.

Peru: La Libertad Department.

##### Etymology.

This species is named after Sofia Carranza Pereyra, daughter of the collector of the type specimens.

#### ﻿ Atopsyche yanachaga sp. nov.

Taxon classificationAnimaliaTrichopteraHydrobiosidae

E0838A90-8624-5782-A93D-0A7AF5A316F7

https://zoobank.org/5C5ACB35-C6D8-46A1-8498-C4C5EA048F69

Figs 14, 15

##### Type material.

***Holotype*.** Peru • 1♂; Pasco, Yanachaga-Chemillén NP, Quebrada San Alberto at Refugio El Cedro; 10.5452°S, 75.3578°W, 2421 m a.s.l.; 07 May 2011; C. Carranza, M. Alvarado, L. Figueroa leg.; light trap; MUSM-ENT-320971 (MUSM). ***Paratypes*.** Peru • 1♂; same data as the holotype, but 12 Nov. 2010; C. Carranza, J. Peralta leg. (MUSM) • 1♂; same data as the holotype, but 13 Jun. 2010; E. Rázuri, C. Carranza leg. (UMSP).

##### Diagnosis.

*Atopsyche yanachaga* is the second species related to the species traditionally included in the subgenus Dolochorema. It is most similar to *A. major* Schmid, 1989 (Bolivia). In lateral view, both species have an inferior appendage slightly longer than high, a low and quadrate segment IX, and relatively narrow, apically rounded parapods. However, certain details differ in these structures. The posteroventral margin of the first segment of the inferior appendage is more pronounced in the new species, whereas in *A. major*, the posterodorsal margin is more pronounced. Additionally, the second segment of the inferior appendage is longer. Furthermore, in the new species, the anterior margin of segment IX is shorter than the posterior margin, whereas both margins are of equal size in *A. major*. Finally, the parapods of the new species is slightly bent downwards at the base and have a sinuous dorsal margin, whereas those of *A. major* are straight, with a sharp point and a rounded indentation on the apical half of the dorsal margin.

##### Description.

**Figure 14.**
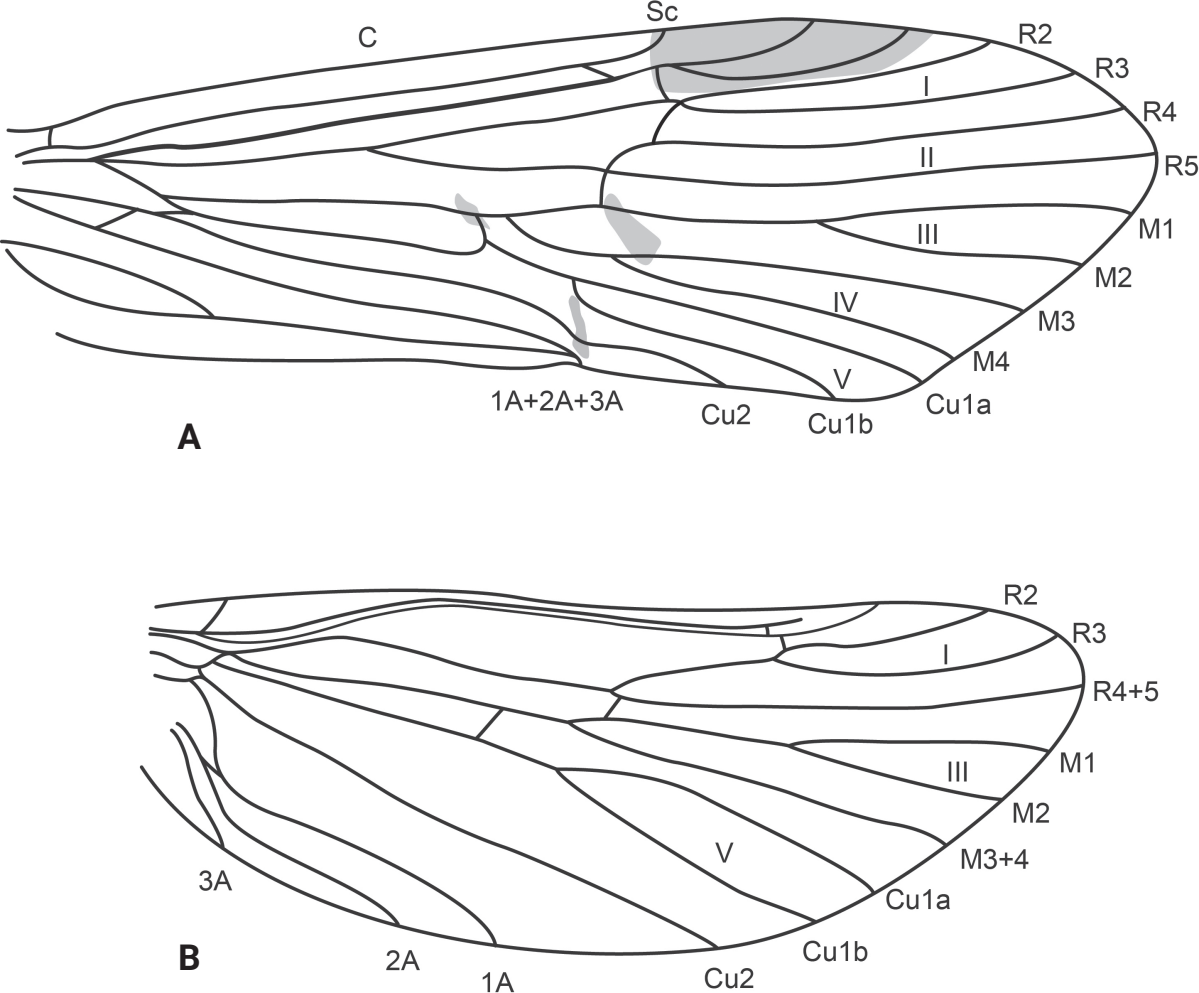
*Atopsyche yanachaga* sp. nov., wing venation. **A.** Forewing; **B.** Hindwing.

**Adult**. Forewing length: male (9.5 mm, *n* = 3). Body and wings brown. Forewing with scattered brown and straw-colored setae along major longitudinal veins; wing membrane with brown and straw-colored setae. Wing venation as in Fig. 14. Sterna III and IV without glands; sternum V with a pair of long, membranous glands; process on sternum VI slightly longer than its segment, straight, bearing fine setae on its basal half and spine-like setae on its apical half, process on sternum VII very short, subtriangular, bearing spine-like setae.

**Figure 15.**
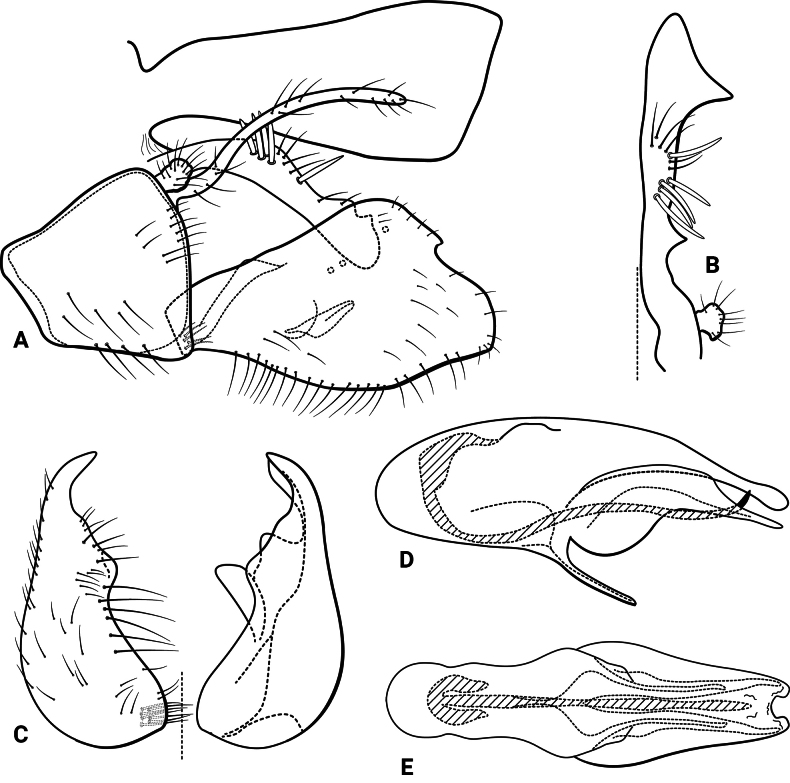
*Atopsyche yanachaga* sp. nov., male genitalia. **A.** Segments IX and X, lateral; **B.** Right parapod and preanal appendage, dorsal; **C.** Inferior appendages, ventral; **D.** Phallic apparatus, lateral; **E.** Phallic apparatus, dorsal.

***Male genitalia*.** Segment IX, in lateral view, quadrangular, posterior margin almost twice the length of the anterior margin, both margins almost straight (Fig. 15A). Parapod, in lateral view, elongate, sausage-shaped, slightly broader mesally and apically, slightly bent ventrad, dorsal surface with a few setae and 5 peg-like setae mesally, apex rounded (Fig. 15A); in dorsal view, elongate, slightly flattened, lateral margin sinuous with acute projections basally and subapically, mesal margin straight, setae on dorsal surface and peg-like setae mesally, apex subacute (Fig. 15B). Filipod long, slender, with setae of different size along length, apex rounded (Fig. 15A). Preanal appendage short, rounded, setose (Fig. 15A). First segment of inferior appendage, in lateral view, quadrangular, ventral margin slightly convex mesally, dorsal margin convex subapically (forming a broad, bilobed process in ventral view), posterior margin rounded, with setae on margins and lateral surface, mesal surface with a group of ~8 long, thick spine-like setae basally and three short, spine-like setae mesally (only 2 spine-like setae on paratype) (Fig. 15A); in ventral view, subtriangular, setose, lateral margin convex, mesal margin sinuous (Fig. 15C); second segment of inferior appendage, in lateral view, triangular, with a few minute setae apically, dorsal margin straight, ventral margin convex, apex narrowly rounded (Fig. 15A); in ventral view, thumb-shaped, apex rounded (Fig. 15C). Proctiger, in lateral view, narrow basally, slightly wider apically, membranous, apex truncate (Fig. 15A). Phallic apparatus complex; phallotheca broadly rounded basally, phallic apodeme indiscernible; with ventral process articulating with inferior appendages, narrow, same width throughout its length; ventrolateral branches of phallotheca present, leaf-shaped, as long as posterior half of phallotheca, acute apically; dorsal process of phallotheca absent; posterior section of phallotheca, in lateral view, broad basally, tapering towards apex, directed slightly posteroventrad, apex rounded, capitate (Fig. 15D); in dorsal view, with a shallow notch mesally (Fig. 15E); phallic spine elongate, stout, spine-like structure, with a strong convex curvature near base, then slightly sinuous (Fig. 15D); in dorsal view, apex acute (Fig. 15E).

##### Distribution.

Peru: Pasco Department.

##### Etymology.

*Atopsyche yanachaga* is named after the Yanachaga-Chemillén National Park, where the type locality is situated. *Yanachaga* means black bump in Quechua, referring to the dark appearance of these mountains during sunsets on sunny days.

### ﻿Species re-illustrations

#### ﻿ Atopsyche (Dolochorema) bispinosa

Taxon classificationAnimaliaTrichopteraHydrobiosidae

Schmid, 1989

DE8EF66E-A247-5A6E-8462-D6A0D9743618

**Figure 16.**
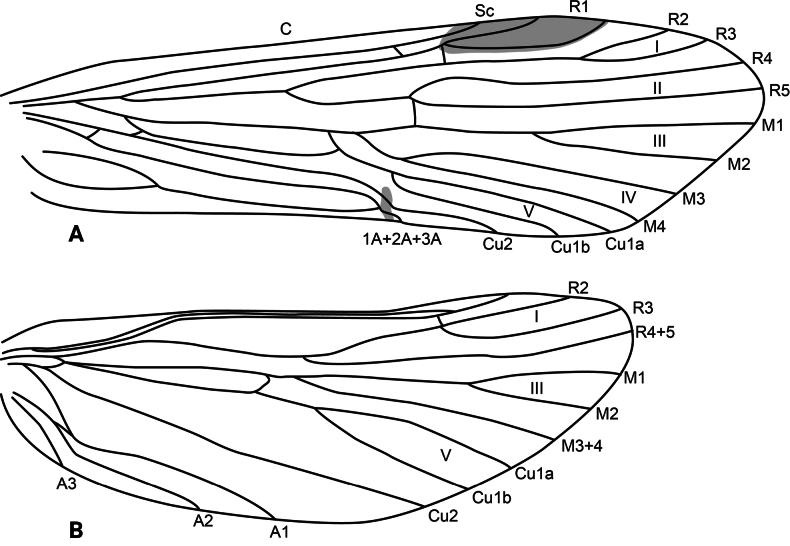
*Atopsyche bispinosa* Schmid, 1989, wing venation. **A.** Forewing; **B.** Hindwing.

**Figure 17.**
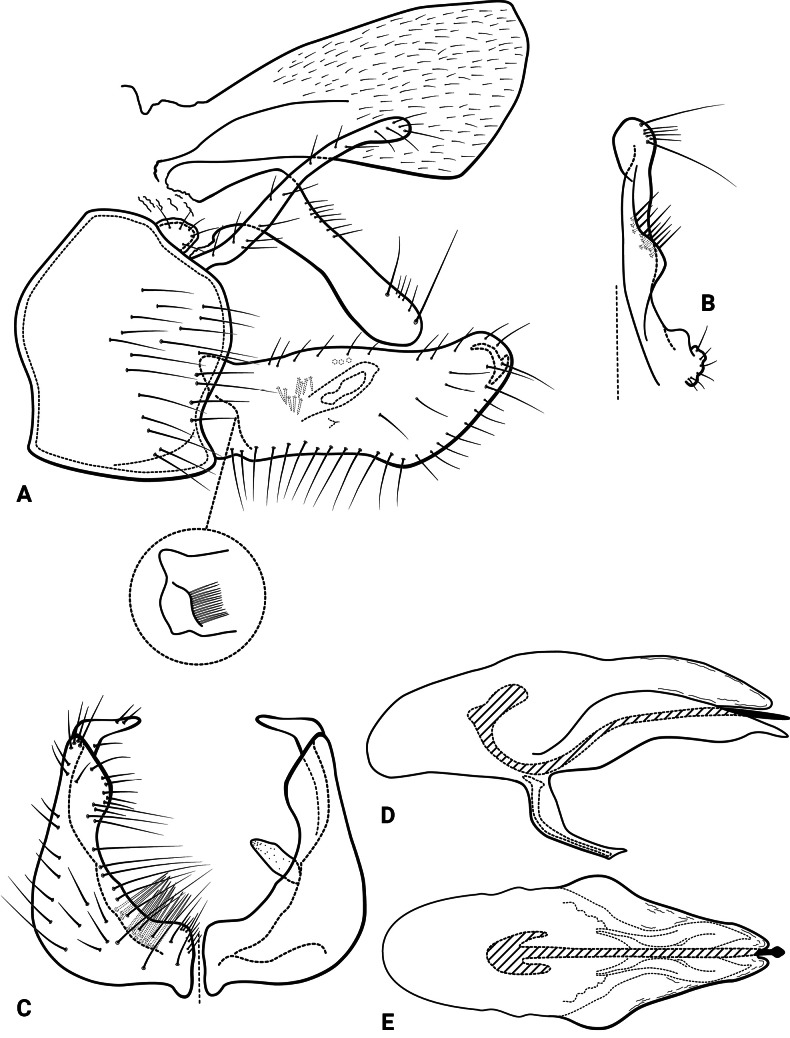
*Atopsyche bispinosa* Schmid, 1989, male genitalia. **A.** Segments IX and X, lateral (inset: group of long setae on mesal surface); **B.** Right parapod and preanal appendage, dorsal; **C.** Inferior appendages and segment IX, ventral (inset: second segment of inferior appendage); **D.** Phallic apparatus, lateral; **E.** Phallic apparatus, dorsal.

Fig. 16(wing venation), Fig. 17 (male genitalia)

Atopsyche (Dolochorema) bispinosa Schmid, 1989: 120 [Type locality: Bolivia, Dpto. La Paz, Unduavi-Coroico; NMNH; ♂]. Angrisano 1999: 27 [checklist].

##### Material examined.

***Paratype*.** Bolivia • ♂; La Paz, Unduavi/Coroico; 2500 m a.s.l.; 19–25 Nov. 1984; L. E. Peña G. leg.; USNM.

##### Distribution.

Bolivia.

#### ﻿ Atopsyche (Dolochorema) irregularis

Taxon classificationAnimaliaTrichopteraHydrobiosidae

(Banks, 1913)

F4AE598E-C4F3-51AC-9F2A-0940629F4ED8

**Figure 18.**
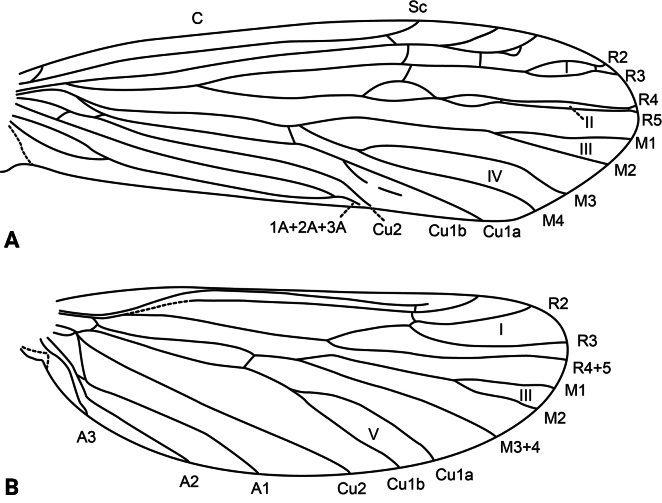
*Atopsyche irregularis* (Banks, 1913), wing venation. **A.** Forewing; **B.** Hindwing.

**Figure 19.**
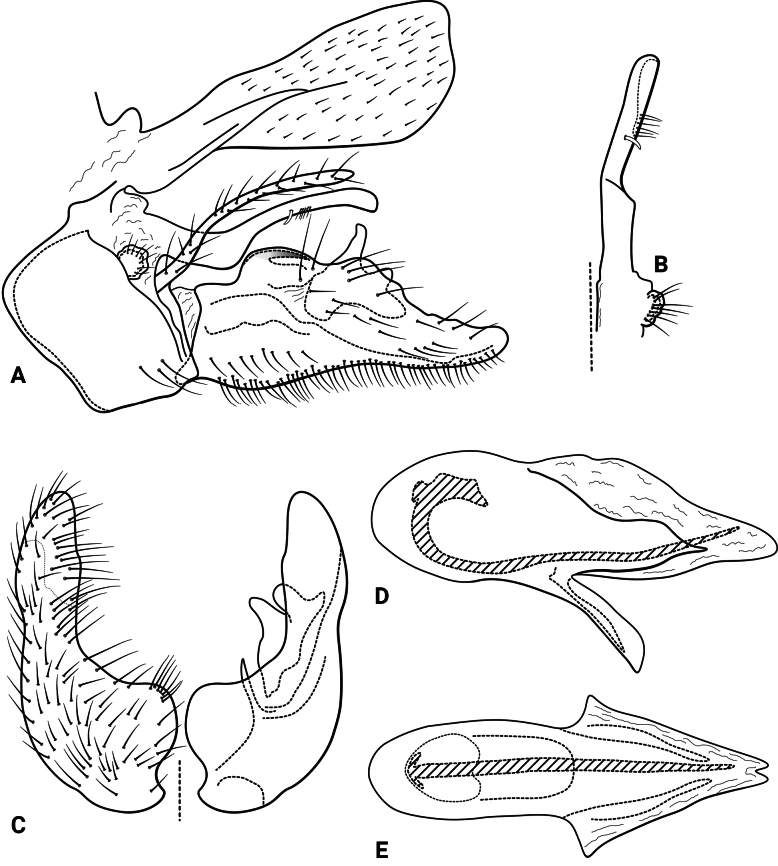
*Atopsyche irregularis* (Banks, 1913), male genitalia. **A.** Segments IX and X, lateral; **B.** Right parapod and preanal appendage, dorsal; **C.** Inferior appendages and segment IX, ventral (inset: second segment of inferior appendage); **D.** Phallic apparatus, lateral; **E.** Phallic apparatus, dorsal.

Fig. 18(wing venation), Fig. 19 (male genitalia)

Atopsyche (Dolochorema) irregularis Banks, 1913: 240 [Type locality: Peru, S.E., Cuzco; MCZ; ♂; in Dolochorema]. Ross 1956: 125 [♂]. Schmid 1989: 160 [to Atopsyche].

##### Material examined.

***Holotype*.** Peru • ♂; Cuzco, S. E. Peru; 2300 m a.s.l.; Anton Heinrich Hermann Fassl leg.; Type 11785; MCZ.

##### Distribution.

Peru.

##### Remarks.

*Atopsyche irregularis* was the first species described in the genus *Dolochorema* (Banks, 1913), and later synonymized with *Atopsyche* (Schmid 1989). It has only been recorded from the Cuzco Department in Peru.

#### ﻿ Atopsyche (Dolochorema) major

Taxon classificationAnimaliaTrichopteraHydrobiosidae

Schmid, 1989

A8045900-F681-565F-AC8A-2F27B2F63BFF

**Figure 20.**
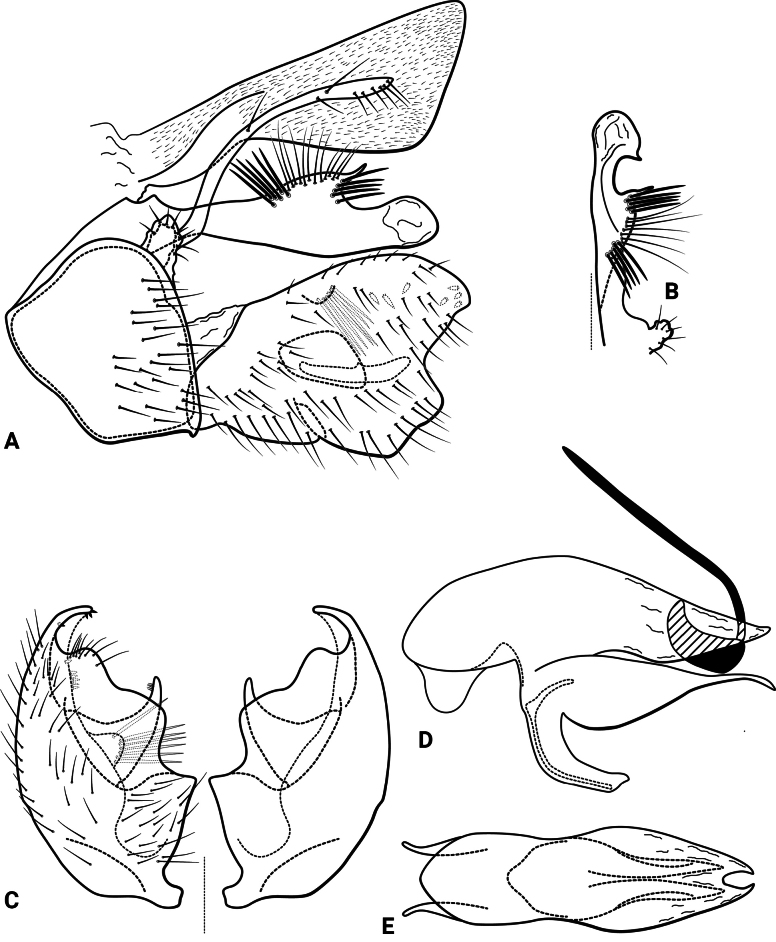
*Atopsyche major* Schmid, 1989, male genitalia. **A.** Segments IX and X, lateral; **B.** Right parapod and preanal appendage, dorsal; **C.** Inferior appendages and segment IX, ventral; **D.** Phallic apparatus, lateral; **E.** Phallic apparatus, dorsal.

Fig. 20 (male genitalia)

Atopsyche (Dolochorema) major Schmid, 1989: 126 [Type locality: Bolivia, Prov. La Paz, Rio Zongo; NMNH; ♂]. Angrisano 1999: 27 [checklist].

##### Material examined.

***Holotype*.** Bolivia • ♂; La Paz, Tarata, Río Zongo; 3200 m a.s.l.; 24–30 Oct. 1984; L. E. Peña G. leg.; USNM.

##### Distribution.

Bolivia.

### ﻿New species records

#### ﻿ Atopsyche (Atopsaura) callosa

Taxon classificationAnimaliaTrichopteraHydrobiosidae

(Navás, 1924)

6C3F6F09-B0DE-5CD4-BC5F-6661B93E89F4

Atopsyche (Atopsaura) callosa Navás, 1924: 78 [Type locality: Costa Rica; MNHNP; ♂; in Ventrarma]. Flint 1975: 566 [synonymy]. McElravy et al. 1981: 152 [distribution]. Holzenthal 1988: 53 [distribution]. Sykora 1991: 250 [distribution]. Flint 1991: 18 [♂; distribution]. Aguila 1992: 533 [distribution]. Flint 1996: 379 [distribution]. Angrisano 1999: 26 [checklist]. Muñoz-Quesada 2000: 275 [checklist]. Holzenthal and Cressa 2002: 134 [distribution]. Rueda Martín 2006: 58 [larva; pupa; distribution]. Isa Miranda and Rueda Martín 2014: 199 [distribution]. Armitage et al. 2015: 8 [checklist]. Armitage and Cornejo 2015: 197 [checklist].
Atopsyche (Atopsaura) alconura Ross, 1953: 288 [Type locality: Peru, Dpto. Cuzco, Pcia. Paucartambo, Cosnipata Valley; INHS; ♂]. Flint 1975: 566 [to synonymy].
Atopsyche (Atopsaura) schmidi Denning, 1965: 267 [Type locality: Costa Rica, San Jose; CAS; ♂]. Flint 1975: 566 [to synonymy].

##### Material examined.

Peru • 2♂24♀; Pasco, small creek in Yanachaga-Chemillén NP buffer zone, San Daniel sector; 10.4278°S, 75.4732°W, 2134 m a.s.l.; 16 Nov. 2010; C. Carranza, J. Peralta leg.; light trap; MUSM. • 1♂6♀; same data as preceding, but 17 Nov. 2010; MUSM.

##### Distribution.

Argentina, Bolivia, Colombia, Costa Rica, Ecuador, Panama, Peru (Cuzco Department, Pasco Department), Venezuela.

##### Remarks.

*Atopsyche callosa* is one of the most widely distributed species in the genus, recorded from Costa Rica to Argentina (Holzenthal and Calor 2017). In Peru, it has previously been reported from Cuzco (Flint 1996). In this study, we provide the first record of *A. callosa* from Pasco.

#### ﻿ Atopsyche (Atopsyche) explanata

Taxon classificationAnimaliaTrichopteraHydrobiosidae

Ross, 1953

9B567C89-5388-5D22-B255-D364D7934DC9

Atopsyche (Atopsyche) explanata Ross, 1953: 288 [Type locality: Peru, Dpto. Cuzco, Pcia. Paucartambo, Cosnipata Valley; INHS; ♂].

##### Material examined.

Peru • 5♂; Pasco, small creek in Yanachaga-Chemillén NP buffer zone, San Daniel sector; 10.4278°S, 75.4732°W, 2134 m a.s.l.; 16 Jun. 2010; E. Rázuri, C. Carranza leg.; light trap; MUSM. • 4♂; same data as preceding, but 17 Jun. 2010; MUSM. • 4♂; same data as preceding, but 03 May 2011; C. Carranza, M. Alvarado, L. Figueroa leg.; MUSM. • 3♂; same data as preceding, but 04 May 2011; MUSM. • 1♂; same data as preceding, but 24 Aug. 2015; E. Rázuri, L. Figueroa, B. Portuguez leg.; UMSP.

##### Distribution.

Peru (Cuzco Department, Pasco Department).

##### Remarks.

Ross (1953) described *Atopsyche explanata* from an unspecified locality in the Cosñipata Valley in the Cuzco Department (Peru). It has also been recorded from Puente Unión in the Cosñipata Valley (UMSP; GBIF.org 2025). We record it for the first time from the Pasco Department, approximately 520 km northwest of Puente Unión.

#### ﻿ Atopsyche (Atopsyche) ikonnikovi

Taxon classificationAnimaliaTrichopteraHydrobiosidae

Martynov, 1912

3EDD2084-E8AE-51A0-A9F7-8F85756A3D1B

Atopsyche (Atopsyche) ikonnikovi Martynov, 1912: 34 [Type locality: Peru, La Merced; type depository unknown; ♂]. Ulmer 1913: 384 [distribution]. Ross and King 1952: 195 [♂]. Angrisano 1999: 27 [checklist].

##### Material examined.

Peru: 1♂; Huánuco, Monson [Monzón] Valley, Tingo María; 23 Sep. 1954; E. I. Schlinger, E. S. Ross leg.; CAS.

##### Distribution.

Argentina, Bolivia, Peru (Huánuco Department, Junín Department).

##### Remarks.

This species was originally described from La Merced in central Peru (Martynov 1912). Ulmer (1913) later recorded it from Salta, Argentina, and Angrisano (1999) noted its presence in Bolivia. We now present a new record from Tingo María, located approximately 200 km north of La Merced.

#### ﻿ Atopsyche (Atopsaura) lobosa

Taxon classificationAnimaliaTrichopteraHydrobiosidae

Ross & King, 1952

D61A64C1-835D-5036-8D2C-D5CD7F96F81A

Atopsyche (Atopsaura) lobosa Ross & King, 1952: 200 [Type locality: Bolivia, Incachaca; CMNH; ♂]. Flint 1996: 380 [distribution]. Angrisano 1999: 26 [checklist]. Ríos-Touma et al. 2017: 27 [distribution]. Mey and Ospina-Torres 2018: 24 [distribution]. Rázuri-Gonzales et al. 2025: 27 [distribution].

##### Material examined.

Peru • 1♂; Pasco, small creek in Yanachaga-Chemillén NP buffer zone, San Daniel sector; 10.4278°S, 75.4732°W, 2134 m a.s.l.; 16 Jun. 2010; E. Rázuri, C. Carranza leg.; light trap; MUSM. • 1♀; same data as preceding, but 17 Jun. 2010; MUSM. • 1♂4♀; Pasco, Yanachaga-Chemillén NP, Quebrada San Alberto at Refugio El Cedro; 10.5452°S, 75.3578°W, 2421 m a.s.l.; 27 Aug 2015; E. Rázuri, L. Figueroa, B. Portuguez leg.; light trap; MUSM.

##### Distribution.

Bolivia, Ecuador, Colombia, Peru (Cuzco, Pasco).

##### Remarks.

*Atopsyche lobosa* was originally described from Bolivia, and subsequently also found in Peru (Cuzco Department). Later, Ríos-Touma et al. (2017) recorded it from Ecuador for the first time. Mey and Ospina-Torres (2018) have also recorded it from Colombia. Rázuri-Gonzales et al. (2025) recorded it from the Zamora Chinchipe Province (Ecuador). Here we also record it from the Pasco Department.

#### ﻿ Atopsyche (Atopsaura) puharcocha

Taxon classificationAnimaliaTrichopteraHydrobiosidae

Schmid, 1989

9AA73A42-ECA8-56E1-8ECF-D27F442218BD

Atopsyche (Atopsaura) puharcocha Schmid, 1989: 129 [Type locality: Bolivia, Prov. Cochabamba, Rio Ronquito, rd. to Villa Tunari, Chapare; NMNH; ♂]. Sykora 1991: 150 [distribution]. Flint 1996: 380 [distribution]. Angrisano 1999: 27 [checklist].

##### Material examined.

Peru • 1♂2♀; Pasco, small creek in Yanachaga-Chemillén NP buffer zone, San Daniel sector; 10.4278°S, 75.4732°W, 2134 m a.s.l.; 03 May 2011; C. Carranza, M. Alvarado, L. Figueroa leg.; light trap; MUSM. • 2♀; same data as preceding, but 04 May 2011; MUSM.

##### Distribution.

Bolivia, Ecuador, Peru (Cuzco Department, Pasco Department).

##### Remarks.

This species was first described by Schmid (1989) from Bolivia, and later reported by Sykora (1991) from Ecuador. Flint (1996) recorded it from Puente San Pedro in the Cosñipata Valley (Cuzco Department). In this study, we report it in the San Daniel Sector of the Yanachaga-Chemillén NP (Pasco Department).

#### ﻿ Atopsyche (Atopsaura) tincuracu

Taxon classificationAnimaliaTrichopteraHydrobiosidae

Schmid, 1989

44E2937A-BB34-53FF-8316-FCC99EF6305A

Atopsyche (Atopsaura) tincuracu Schmid, 1989: 130 [Type locality: Bolivia, Prov. La Paz, Unduavi/Coroico; NMNH; ♂]. Angrisano 1999: 27 [checklist].

##### Material examined.

Peru • 1♂1♀; Pasco, Yanachaga-Chemillén NP, Quebrada San Alberto at Refugio El Cedro; 10.5452°S, 75.3578°W, 2421 m a.s.l.; 27 Aug 2015; E. Rázuri, L. Figueroa, B. Portuguez leg.; light trap; MUSM. • 1♂; same data as preceding; UMSP.

##### Distribution.

Bolivia, Peru (Pasco Department).

##### Remarks.

*Atopsyche tincuracu* was originally described from Bolivia. In this study, we record its occurrence in Peru (Pasco Department), approximately 1000 km northwest of the type locality.

#### ﻿ Atopsyche (Atopsyche) vatucra

Taxon classificationAnimaliaTrichopteraHydrobiosidae

Ross, 1953

886EFE66-76D3-5290-A85F-830C4F71B2D8

Atopsyche (Atopsyche) vatucra Ross, 1953: 290 [Type locality: Peru, Dpto. Cuzco, Pcia. Paucartambo, Cosnipata Valley; INHS; ♂]. Flint 1996: 379 [distribution].

##### Material examined.

Peru • 2♂1♀; Pasco, small creek in Yanachaga-Chemillén NP buffer zone, San Daniel sector; 10.4278°S, 75.4732°W, 2134 m a.s.l.; 17 Jun. 2010; E. Rázuri, C. Carranza leg.; light trap; MUSM. • 1♂; same data as preceding, but 16 Nov. 2010; C. Carranza, J. Peralta leg.; MUSM. • 2♂; same data as preceding, but 17 Nov. 2010; MUSM. • 5♂1♀; same data as preceding, but 03 May 2011; C. Carranza, M. Alvarado, L. Figueroa leg.; MUSM. • 5♂2♀; same data as preceding, but 04 May 2011; MUSM.

##### Distribution.

Bolivia, Ecuador, Peru (Cuzco Department, Pasco Department), Venezuela.

##### Remarks.

*Atopsyche vatucra* was originally described from an unspecified locality in the Cosñipata Valley, Cuzco, Peru (Ross 1953), and subsequently reported from additional sites within the valley (Flint 1996). This species has also been documented in Bolivia, Ecuador, and Venezuela (UMSP; GBIF.org 2025). In this study, we report this species from the San Daniel sector in the Yanachaga-Chemillén National Park buffer zone, located approximately 500 km northwest of previously known localities for this species in Peru.

## ﻿Discussion

Before this study, only 12 species of *Atopsyche* were known from Peru, with the most recent description dating back 36 years (Schmid 1989). This paper describes seven new species and records *A. tincuracu* from the country for the first time, bringing the total number of *Atopsyche* in the country to 20 and representing a 35% increase (see Table 1). As a result, the total number of species in the genus rises to 160.

**Table 1.** Updated list of *Atopsyche* species from Peru.

Species	Department	Endemic	Elevation (m a.s.l.)	Source
*A. bicolorata* Schmid, 1958	CU		2800	INHS (GBIF.org 2025)
*A. callosa* (Navás, 1924)	CU, PA		1050–2134	Flint 1996, this study
*A. cedroi* sp. nov.	PA	E	2421	This study
*A. chemillen* sp. nov.	PA	E	2134	This study
*A. corcuerai* sp. nov.	CA	E	2522	This study
*A. explanata* Ross, 1953	CU, PA	E	1600–2134	Ross 1953, this study
*A. huascarani* sp. nov.	AN	E	4412	This study
*A. ikonnikovi* Martynov, 1912	HU, JU		647–750	Martynov 1912, this study
*A. irregularis* (Banks, 1913)	CU	E	2300	Banks 1913
*A. kingi* Ross, 1953	CU		1050–1450	Ross 1953, Flint 1996
*A. lobosa* Ross & King, 1952	CU, PA		1600–2800	Flint 1996, Mey and Ospina-Torres 2018, this study
*A. mancocapac* Schmid, 1989	CU		400	Flint 1996
*A. neotropicalis* Schmid, 1989	CU, MD	E	400–1430	Schmid 1989, Flint 1996
*A. puharcocha* Schmid, 1989	CU, PA		1430–2134	Schmid 1989, Flint 1996, this study
*A. refulioae* sp. nov.	PA	E	2421	This study
*A. sofiae* sp. nov.	LL	E	3482	This study
*A. tincuracu* Schmid, 1989	PA		2421	New country record
*A. ulmeri* Ross, 1953	CU		1050–1430	Ross 1953, Flint 1996
*A. vatucra* Ross, 1953	CU, PA		1050–2134	Ross 1953, Flint 1996, this study
*A. yanachaga* sp. nov.	PA	E	2421	This study

AN: Ancash, CA: Cajamarca, CU: Cuzco, HU: Huánuco, JU: Junín, LL: La Libertad, MD: Madre de Dios, PA: Pasco. INHS: Illinois Natural History Survey, Champaign, Illinois, USA.

Most *Atopsyche* species in Peru are recorded at mid to high elevations (above 1000 m a.s.l.), with only three occurring below this elevation (*A. ikonnikovi*, *A. mancocapac*, and *A. neotropicalis*). Furthermore, *Atopsyche huascarani*, recorded from a glacier-fed stream at 4412 m a.s.l. in the Cordillera Blanca (Ancash Department), represents the highest record of *Atopsyche* across its distributional range. In Peru, most *Atopsyche* occur along the eastern flank of the central and southern Cordillera Oriental (Huánuco, Junín, Pasco, and Cuzco Departments). Three of the new species described in this study inhabit streams on the northern Cordillera Occidental and Cordillera Central (Cajamarca, La Libertad, and Ancash Departments), constituting the first species to be described from these parts of the Andes. Although Flint and Reyes (1991) recorded two *Atopsyche* species from Otuzco and Simbal in La Libertad Department, these were only represented by females and not formally described.

Half of the *Atopsyche* species found in Peru also occur in neighboring countries. Ecuador currently includes 37 species (Rázuri-Gonzales et al. 2025), with only four shared exclusively with Peru. Similarly, Bolivia includes ten species (Holzenthal and Calor 2017), three of which are shared only with Peru. In contrast, Colombia harbors only nine *Atopsyche* species, with only two also found in Peru. These two species, *Atopsyche callosa* and *A. lobosa*, are widely-distributed, occurring from Costa Rica to Argentina and from Colombia to Bolivia, respectively (Holzenthal and Calor 2017). Given the high number of endemic species in Peru (10) and Ecuador (27), along with limited sampling and significant geographic gaps, many more undescribed species of *Atopsyche* are likely to exist in the region.

Five species described in this study were collected from national parks, their buffer zones (Yanachaga-Chemillén and Huascarán National Parks), or private conservation areas (Bosque Cachil). Further autecological and life history studies are needed for *A. huascarani* to better assess its conservation status. This species was described based on only two males and one female specimens, collected from a small creek originating in the glacier-fed Laguna Llaca (fed by the Oschapalca and Ranrapalca Glaciers). Unfortunately, substantial glacier shrinkage has occurred in the area over the past few decades (Racoviteanu et al. 2008; Juřicová and Fratianni 2018), placing this species under considerable threat of extirpation. Caddisflies show a tendency to fly upstream after emergence (Holzenthal et al. 2015) and have low lateral dispersal ability in alpine streams (Finn and Poff 2008), which could prevent *A. huascarani* from dispersing to neighboring valleys, thus limiting gene flow. The nearest valley is more than 3.5 km away, with mountains higher than 700 meters acting as a barrier.

Nine of the 20 species of *Atopsyche* from Peru occur in two streams within the Yanachaga-Chemillén NP and its buffer zone. The creek sampled in the San Daniel Sector was 1.5-meter-wide with a rocky bottom and medium-sized boulders covered in algae and moss, and it was located in a 60-meter-wide forest strip in the buffer zone, adjacent to a cattle pasture. Six species were found in this creek (*A. callosa*, *A. chemillen*, *A. explanata*, *A. lobosa*, *A. puharcocha*, and *A. vatucra*). Of these, only *A. chemillen* was present in all four sampling events (Jun. 2010, Nov. 2010, May 2011, Aug. 2015).

The San Antonio stream in the El Cedro Sector of the Yanachaga-Chemillén NP yielded five *Atopsyche* species (*A. cedroi*, *A. lobosa*, *A. refulioae*, *A. tincuracu*, and *A. yanachaga*), three of which are new to science. Interestingly, only *A. lobosa* was found at both sites, despite their close proximity (only 18 km apart). This suggests a potentially much higher species richness of *Atopsyche* and Trichoptera in general within the park. Spanning both sides of the Cordillera de Yanachaga (a part of the Cordillera Oriental), the park’s broad elevation range (460–3643 m a.s.l.) and multiple river basins likely contribute to this diversity (Instituto Nacional de Recursos Naturales 2006).

## Supplementary Material

XML Treatment for Atopsyche cedroi

XML Treatment for Atopsyche chemillen

XML Treatment for Atopsyche corcuerai

XML Treatment for Atopsyche (Dolochorema) huascarani

XML Treatment for Atopsyche refulioae

XML Treatment for Atopsyche sofiae

XML Treatment for Atopsyche yanachaga

XML Treatment for Atopsyche (Dolochorema) bispinosa

XML Treatment for Atopsyche (Dolochorema) irregularis

XML Treatment for Atopsyche (Dolochorema) major

XML Treatment for Atopsyche (Atopsaura) callosa

XML Treatment for Atopsyche (Atopsyche) explanata

XML Treatment for Atopsyche (Atopsyche) ikonnikovi

XML Treatment for Atopsyche (Atopsaura) lobosa

XML Treatment for Atopsyche (Atopsaura) puharcocha

XML Treatment for Atopsyche (Atopsaura) tincuracu

XML Treatment for Atopsyche (Atopsyche) vatucra

## ﻿Additional information

### Use of AI

No use of AI was reported.

### Author ORCIDs

Ernesto Rázuri-Gonzales https://orcid.org/0000-0002-7554-0816

Ralph W. Holzenthal https://orcid.org/0000-0003-1853-6340

### Data availability

All of the data that support the findings of this study are available in the main text.
